# RecA-dependent or independent recombination of plasmid DNA generates a conflict with the host EcoKI immunity by launching restriction alleviation

**DOI:** 10.1093/nar/gkae243

**Published:** 2024-04-03

**Authors:** Mikhail Skutel, Daria Yanovskaya, Alina Demkina, Aleksandr Shenfeld, Olga Musharova, Konstantin Severinov, Artem Isaev

**Affiliations:** Skolkovo Institute of Science and Technology, Moscow, Russia; Skolkovo Institute of Science and Technology, Moscow, Russia; Moscow Institute of Physics and Technology, Moscow, Russia; Skolkovo Institute of Science and Technology, Moscow, Russia; Shemyakin-Ovchinnikov Institute of Bioorganic Chemistry, Moscow, Russia; Skolkovo Institute of Science and Technology, Moscow, Russia; Skolkovo Institute of Science and Technology, Moscow, Russia; Institute of Molecular Genetics, National Research Center Kurchatov Institute, Moscow, Russia; Waksman Institute of Microbiology, Piscataway, USA; Institute of Gene Biology, Russian Academy of Sciences, Moscow, Russia; Skolkovo Institute of Science and Technology, Moscow, Russia

## Abstract

Bacterial defence systems are tightly regulated to avoid autoimmunity. In Type I restriction–modification (R–M) systems, a specific mechanism called restriction alleviation (RA) controls the activity of the restriction module. In the case of the *Escherichia coli* Type I R–M system EcoKI, RA proceeds through ClpXP-mediated proteolysis of restriction complexes bound to non-methylated sites that appear after replication or reparation of host DNA. Here, we show that RA is also induced in the presence of plasmids carrying EcoKI recognition sites, a phenomenon we refer to as plasmid-induced RA. Further, we show that the anti-restriction behavior of plasmid-borne non-conjugative transposons such as Tn5053, previously attributed to their *ardD* loci, is due to plasmid-induced RA. Plasmids carrying both EcoKI and Chi sites induce RA in RecA- and RecBCD-dependent manner. However, inactivation of both RecA and RecBCD restores RA, indicating that there exists an alternative, RecA-independent, homologous recombination pathway that is blocked in the presence of RecBCD. Indeed, plasmid-induced RA in a RecBCD-deficient background does not depend on the presence of Chi sites. We propose that processing of random dsDNA breaks in plasmid DNA via homologous recombination generates non-methylated EcoKI sites, which attract EcoKI restriction complexes channeling them for ClpXP-mediated proteolysis.

## Graphical Abstract

**Graphical Abstract:**
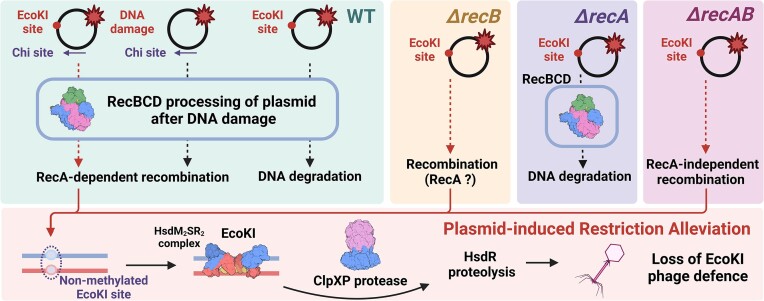

## Introduction

Bacteria and phages are locked in a constant arms race that generates an enormous variety of defensive and offensive strategies (1–3). The anti-phage arsenal of bacteria should be ‘balanced’ to avoid autoimmunity (4,5). To lower the negative costs, defence genes are tightly regulated (6–8), and are often induced only at conditions of higher cell density, which favor phage propagation (9,10). In addition, ‘inherent brake’ mechanisms such as ring nucleases in Type III CRISPR-Cas or Trip13-like ATPases in Type III CBASS systems prevent false activation of defence systems by repressing secondary messenger signaling (11,12). In the absence of phage pressure, defence genes can be lost to decrease fitness costs associated with their toxicity (13,14). As a consequence, anti-phage immunity systems represent the major source of variability between closely related bacterial strains (15,16).

Defensive activity of some Type I restriction–modification (R–M) systems is controlled through a restriction alleviation (RA) mechanism. Type I R–M systems encode three subunits: HsdM (methyltransferase), HsdS (site recognition) and HsdR (endonuclease and ATPase that powers DNA translocation). The restriction enzyme is a heteropentameric M_2_S_1_R_2_ complex that also possesses the methylation activity; the M_2_S_1_ complex is capable of methylation only (17,18). The prototypical Type I system EcoKI recognizes asymmetric bipartite DNA sites AACNNNNNNGTGC, which are methylated at adenines in both DNA strands at the underlined positions. Upon recognition of a non-methylated site, HsdR subunits start translocating DNA, which is looped through the DNA-bound restriction complex, resulting in ‘rabbit ear’ structures (19,20). The DNA cleavage reaction occurs when translocation initiated by two neighboring complexes results in HsdR subunits collision, upon which each nuclease introduces a nick, jointly producing a double-stranded DNA break (19–21). Other obstacles that interfere with DNA translocation, such as replication forks, can also activate the DNA cleavage reaction (22). Hemi-methylated sites that appear after replication are recognized by methylation complexes which add the missing methyl group. However, replication fork stalling followed by recombinational repair between two newly synthesized non-methylated DNA strands could result in the appearance of non-methylated sites that will be subject to restriction and thus damaging to the host (23).

RA is one of the signature phenotypes observed upon UV irradiation of *Escherichia coli* (24,25). Depending on the irradiation dose, *E. coli* cells harboring EcoKI temporarily lose, partially or completely, protection against phage *λ* infection (24–26). A similar behavior is observed when cells are exposed to 2-aminopurine (2-AP), a chemical agent that is incorporated in DNA instead of adenine but is not subject to methylation (27). Based on these observations, it was suggested that RA allows cells to avoid cleavage of non-methylated EcoKI sites in host DNA that appear after replication/reparation and accumulate upon DNA damage. Later studies identified that RA is mediated by the ClpXP protease, which degrades the HsdR endonuclease (28,29). *In vivo* and *in vitro* assays showed that HsdR becomes available for proteolysis during the translocation stage (30,31).

Clearly, RA should not be activated during phage infection. While the exact mechanism is unknown, it can be speculated that the time of translocation along viral DNA is shorter than that on host DNA. All EcoKI sites in incoming phage DNA are non-methylated and thus the time from non-methylated EcoKI site recognition to collision with another complex bound to non-methylated site is short. In contrast, generation of non-methylated sites in EcoKI^+^ host DNA is a random and likely not efficient process, possibly requiring DNA damage events. Thus, the restriction enzyme would spend a much longer time in a proteolysis-prone translocation state before it encounters another complex bound to a different non-methylated EcoKI site. It was also proposed that the difference in the condensation state between the phage and the host DNA might play a role (32). The ClpXP-dependent RA was shown for EcoKI and EcoA systems (28). In the case of EcoR124I it was proposed that RA-like behavior is mediated by the stability of the restriction complex, since HsdR (A957V) mutation increasing the rate of translocation initiation events was shown to produce an RA-deficient phenotype (33–35). Whether other modification-dependent immunity systems rely on proteolytic control of their activity remains to be determined.

Phages and mobile genetic elements developed multiple strategies to cope with R–M systems, from avoidance of recognition sites to extensive modification of their DNA and expression of anti-restriction proteins (36–38). Examples of the latter strategy include a T7 phage DNA mimic protein Ocr, which competes with DNA for interaction with Type I restriction complexes, and T3 phage S-adenosylmethionine-lyase, which depletes SAM, an essential co-factor for Type I restriction (39–43). The P1 phage injects capsid-loaded DarA and DarB proteins that inactivate different Type I R–M systems through a mechanism that has not been defined (44,45). Many conjugative plasmids and other mobile elements encode anti-restriction Ard (Alleviation of restriction of DNA) proteins. ArdA is a DNA mimic (46); ArdB inhibits Type I R–M *in vivo* through an unknown mechanism (47); ArdC contains an ssDNA binding domain and may protect single-stranded DNA entering cells during conjugation (48). Plasmids carrying some non-conjugative transposons, e.g. Tn5053, also suppress the EcoKI defence (49). A putative gene *ardD* encoded in the anti-sense strand of the transposase gene *tniA* was proposed to be responsible for this phenotype (50). In contrast to other Ard mechanisms, the *ardD* anti-restriction was dependent on ClpXP, suggesting that Tn5053 can manipulate EcoKI activity through an RA-like mechanism (51,52).

Here, we report that ClpXP-dependent RA of the EcoKI system is activated by the presence of plasmids carrying EcoKI and Chi sites. The same plasmid-induced RA mechanism is operational in cells carrying plasmids with cloned non-conjugative transposons. We therefore submit that the putative *ardD* transposon gene that was proposed to be responsible for RA is likely non-existent. The simultaneous presence of plasmid-borne EcoKI and Chi sites in EcoKI^+^ cells promotes RA through RecBCD-dependent recombination. In cells lacking both RecA and RecBCD, plasmid-induced RA that no longer depends on the presence of Chi sites is observed, suggesting the existence of an alternative, RecA-independent recombination pathway that becomes activated when dsDNA ends are not occupied by RecBCD.

## Materials and methods

### Bacterial strains, plasmids and phages

All bacterial strains, plasmids, and phages used in this study are listed in Supplementary Table S1. The majority of experiments were performed with *E. coli* AB1157 strain encoding EcoKI system on a chromosome. An isogenic EcoKI^−^ strain AB1157*ΔhsdM* was used as a control for the phage infection experiments to estimate phage titer. *λ_vir_* is an obligatory lytic mutant of the phage *λ*, while T7*Δ0.3* lacks *0.3* gene encoding anti-restriction protein Ocr and thus is sensitive to EcoKI defence. Both phages were produced on AB1157*ΔhsdM* and thus lacked EcoKI methylation.

Bacterial cultures were routinely propagated in LB medium (Lysogeny Broth: 10 g/L NaCl, 10 g/L Tryptone, 5 g/L Yest Extract) at 37°C with appropriate antibiotics. To produce T7*Δ0.3* phage stock, an overnight bacterial culture was diluted 100-fold in LB and incubated at 37°C until OD_600_ ∼0.6. The culture was infected with phage at low MOI (multiplicity of infection) and further incubated overnight at 37°C. The resulting lysate was purified from cell debris by centrifugation (6000g, 10 min, 4°C) and treated with chloroform (10 μl per 1 ml of lysate). The resulting phage stock was stored at +4°C. *λ_vir_* lysate was produced on a solid medium plate. An overnight culture was diluted 100-fold in 0.6% LB agar supplemented with 0.2% maltose and 10 mM MgCl_2_, mixed with 10 μl of a high-titer phage *λ_vir_* stock (∼10^10^ pfu/ml) and poured on Petri dishes with solidified 1.2% LB agar. After overnight incubation at 37°C, the top agar was scraped from plates and centrifuged (10,000g, 10 min, 4°C). The supernatant was processed as described above.

### Plasmid construction

Plasmids used in the study are listed in Supplementary Table S1. All primers are listed in Supplementary Table S2. Dar system genes were amplified by PCR with Q5 (NEB), or CloneAmp (TakaraBio) DNA polymerase from P1 genomic DNA, gel-purified with GeneJET Gel Extraction Kit (Thermo Scientific) and ligated into EcoRI linearized pBAD L24 backbone (42), using HiFi DNA Assembly master mix or GA master mix (NEB). Point mutagenesis of Chi and EcoKI sites in pRA and pUC-Tn5053, as well as deletion of *ardD* locus in pUC-Tn5053 was carried with Q5-site directed mutagenesis kit (NEB), CloneAmp (TakaraBio) was used instead of the kit-provided Q5 polymerase for the PCR with pUC-Tn5053. For the expression of *λ gam*, *λ* Red (*exo*+ *beta* genes), or *λ* Red *and* Gam we used pACBSR plasmid from the ‘gene doctoring’ system (53). The plasmid was modified to remove I-SceI gene, yet to retain *araBAD* promoter controlling *λ gam + exo + beta* expression, and then *λ gam* or *λ exo + beta* was further removed via Q5-site directed mutagenesis kit (NEB). Commercial chemocompetent Xl1-Blue cells (Evrogen) were used for transformation. Plasmids were routinely isolated with GeneJET Plasmid Miniprep kit (Thermo Scientific) or Monarch Plasmid Miniprep kit (NEB). All constructs were verified with Sanger sequencing (Evrogen).

### Chromosomal deletions transfer via P1 transduction

The transfer of genetic material from the donor strain to the recipient was carried out using generalized transduction with the P1_vir_ phage. KEIO collection strains (54) were used as donors for the transfer of kanamycin-resistance cassette substituting non-essential genes of interest in the genome of AB1157 recipient. Lysate of P1*_vir_* phage was produced on the KEIO donor strains grown in LB supplemented with 10 mM CaCl_2_ and 5 mM MgSO_4_. For transduction, 1.5 ml of the recipient strain overnight culture was harvested by centrifugation (6000g, 10 min, 4°C) and resuspended in a 700 μl of mQ supplemented with 10 mM CaCl_2_ and 5 mM MgSO_4_. 100 μl aliquots were mixed with various amounts (1–10–100 μl) of P1*vir* lysate (∼5 × 10^10^ pfu/ml). An aliquot of recipient cells without the phage was used as a negative control. For adsorption of phage particles, aliquots were incubated with recipient cells at 37°C for 30 min, followed by dilution in 1 ml of LB with the addition of 200 mM sodium citrate to prevent secondary adsorption of phage particles and re-infection. Next, the cells were incubated for 1 hour at 37°C on a shaker. The cells were harvested by centrifugation and plated on 1.2% LB agar supplemented with antibiotic and 200 mM sodium citrate. The phage stock was also plated on a control plate supplemented with antibiotic as a control for the surviving donor cells. After overnight incubation at 37°C, the resulting colonies were tested by PCR for the presence of a replacement of target gene with the kanamycin resistance marker. PCR-positive clones were re-streaked at least 3 times on 200 mM sodium citrate plates with kanamycin to completely remove phage particles. To construct double deletions, kanamycin resistance marker was removed through Flp-mediated recombination with pCP20. Electrocompetent cells were transformed with pCP20 and plated at 30°C with ampicillin (pCP20 selection) and kanamycin (selective marker for the gene-of-interest). To initiate recombination/pCP20 loss, a single colony was inoculated in 10 ml LB, grown overnight at 42°C without antibiotics and plated at 30°C. The next day, single colonies were resuspended in 10 μl of LB and plated on kanamycin (37°C), ampicillin (30°C) and without antibiotics (37°C). Colonies that showed growth on the plate without antibiotics, but not on kanamycin and ampicillin plates were further PCR-verified for the removal of kanamycin resistance marker in the gene-of-interest locus. A second round of transduction was performed as described above.

### Efficiency of plating (EOP) assay

The titer of phage particles in lysates and efficiency of EoKI defence were determined using the double layer agar method. An overnight culture of bacteria was diluted 100-fold and mixed with 0.6% LB agar, supplemented with required antibiotics and optionally with 0.2% maltose and 10 mM MgCl_2_ for the phage *λ* infection, followed by plating on a pre-cast 1.2% LB agar plates. 0.2% l-arabinose was used to induce transcription from the pBAD and pACBSR vector. For the experiments with pACBSR vectors, overnight culture was also grown in the presence of 0.2% l-arabinose. 10 μl serial 10-fold dilutions of phage stock were pipetted on the top agar and left until dry. After overnight incubation at 37°C, the number of phage plaques was counted, and the titer was determined. The fold protection was calculated as the ratio of the phage titer obtained on the lawn of cells without active defense system (AB1157*ΔhsdM* or its double-deletion derivatives) to the phage titer on the lawn of experimental culture. All EOP experiments were carried out in biological triplicates.

### Liquid culture infectoin dynamics

An overnight culture of bacteria was diluted 100-fold in LB medium supplemented with antibiotics and additives required for the phage infection and incubated at 37°C with shaking until the optical density of the culture (OD_600_) reached ∼0.6. If required, 0.2% l-arabinose was preliminary added at OD_600_ ∼0.3. Next, 200 μl aliquots were transferred to the 96-well plates (Costar) and infected with phages at desired MOI. Optical density was monitored for 20 h using an EnSpire Multimode Plate Reader (PerkinElmer, USA) at 37°C.

### Efficiency of transformation (EOT) assay

To produce EcoKI methylated variant, plasmids were propagated on AB1157, while AB1157*ΔhsdM* was used to produce non-methylated variant. Plasmids were purified from 2 ml of overnight culture with GeneJET Plasmid Miniprep kit (Thermo Scientific) or Monarch Plasmid Miniprep kit (NEB) and the concentration was measured using Qubit dsDNA Broad Range Assay Kit on the Qubit 3.0 Fluorometer (Invitrogen). The equimolar amount of each plasmid (24 fmol) was used for the transformation of 50 μl chemocompetent cells prepared according to Inoue protocol (55). Colonies were counted automatically on a colony counter Scan 1200 (Interscience). M+/M– value was calculated as the ratio between the number of colonies obtained with the methylated plasmid relative to the non-methylated one. All EOT experiments were performed in biological triplicates.

### Plasmids Illumina sequencing

Plasmid DNA was purified with GeneJET Plasmid Miniprep kit (Thermo Scientific). DNA libraries were prepared in accordance with a standard protocol and sequenced on MiniSeq platform (Illumina, USA) with paired-end 150 cycles (75 + 75). Plasmid assembly was performed using SPAdes implemented in Unicycler (56). Plasmid origins were determined based on blastn search.

### Mass-spectrometry

The relative amount of HsdR protein was determined in cell lysates via mass-spectrometry. The overnight culture was diluted 100-fold in 10m ml of LB media and incubated with antibiotics at 37°C with constant shaking until the OD_600_ ∼0.6. The cells were then harvested by centrifugation (6000g, 10 min, 4°C) and resuspended in 200 μl of PBS buffer (Sigma-Aldrich). Cells were lysed by sonication on ice with a 10 second pulse and 40% power using Q500 sonicator with 0.2 cm sonotrode (Qsonica). Cell debris was removed by centrifugation at 4°C for 30 min at a speed of 20 000g. The resulting supernatant was further examined for the HsdR abundance using label-free protein quantification, as described (57). LFQ protein counts for HsdR and HsdM were normalized to the total protein counts.

### Evaluation of the SOS response and flow cytometry

To measure activation of the SOS response, strains MP062 and MP074 (isogenic to MP062, but with *recA* deletion) encoding a chromosomal variant of the YFP reporter under control of *sulA* promoter, were used (5). Culture was grown in LB till OD_600_ ∼0.6 and then 200 μl of culture was transferred to 96-well plate (Costar) and incubation was continued at 37°C in EnSpire Multimode Plate Reader (PerkinElmer). Culture growth was monitored by OD_600_, while YFP signal was excited at a wavelength of 513 nm and detected at 530 nm. YFP fluorescence signal was normalized to the OD value at each time point.

To determine SOS response signal from individual cells, MP062 strain culture was used for the flow cytometry (5). *ΔrecA* MP062 derivative MP074 was used as a negative control for SOS response induction. Bacterial culture started from individual colony was grown overnight at 37°C in LB. Fresh overnight cultures were diluted 100-fold in 10 ml 0.22 μm filtered M9 medium (1xM9 salts, 2 mM MgSO_4_, 0.1mM CaCl_2_) supplemented with 5% LB broth and required antibiotics and grown till exponential phase (OD_600_ ∼ 0.6). As positive control for SOS response activation, cells were treated with 2.5 μg/ml Nalidixic acid at OD_600_ ∼0.1. Measurement of YFP fluorescence was performed with CytoFLEX cytometer (Beckman Coulter) in the FITC channel. 100 000 events were collected for each sample in three biological replicates. Data was analyzed and visualized in FlowJo v10. Single cell events were selected based on the scatter plot distribution of FSC-A/FSC-H values. To quantify SOS signal, YFP expressing cells were gated for FITC signal.

## Results

### Plasmids containing fragments of P1 phage *dar* locus inhibit the EcoKI defence

**Figure 1.**
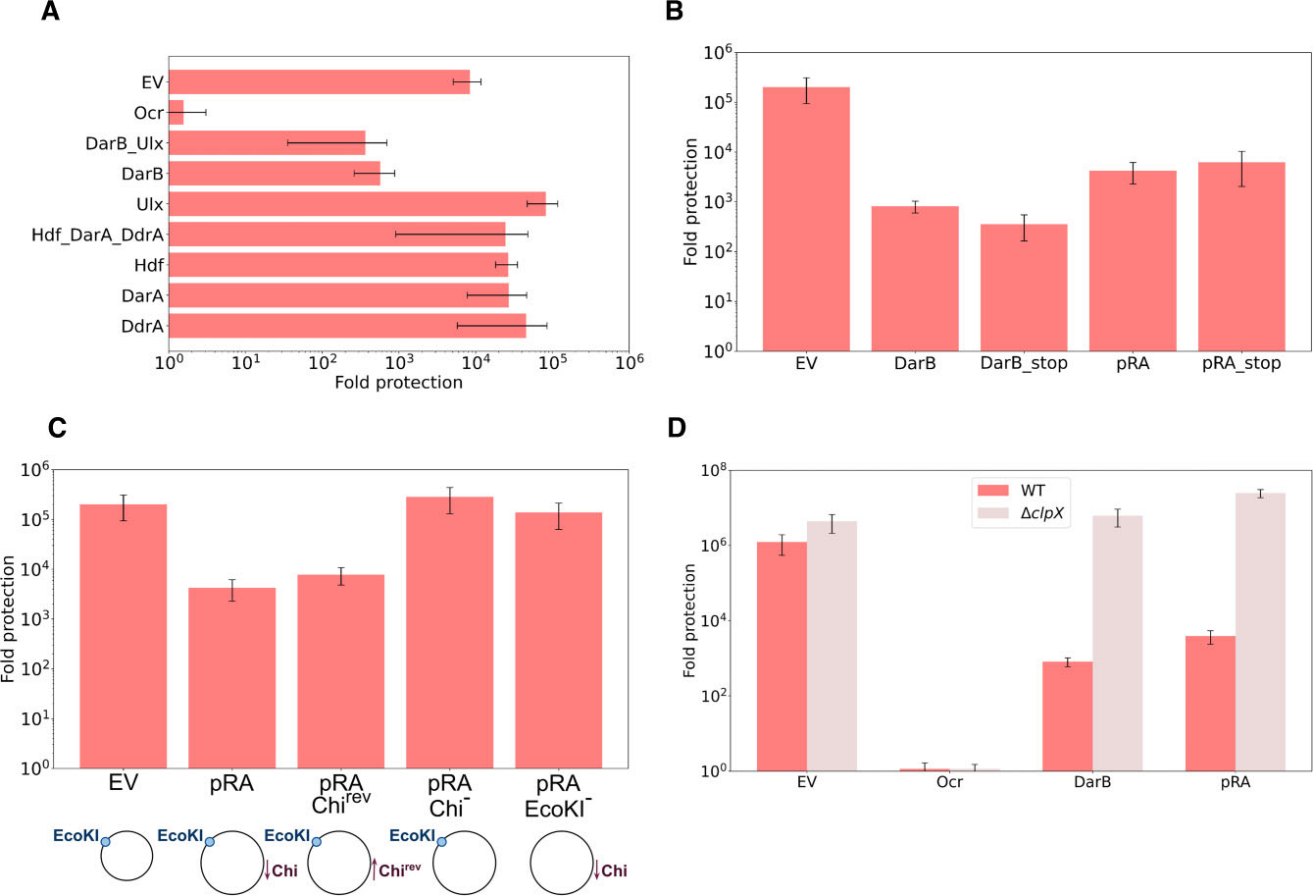
Plasmids with Chi and EcoKI sites alleviate EcoKI restriction in a ClpXP-dependent manner. Fold protection was calculated as a ratio of *λ_vir_* phage titer on EcoKI^−^ AB1157*ΔhsdM* cell lawns to that on the lawns of EcoKI^+^ AB1157 transformed with indicated plasmids. Cells carrying a T7 EcoKI inhibitor Ocr (‘Ocr’) expression plasmid or the pBAD empty vector (‘EV’) served as controls. (**A**) Protection of cells carrying plasmids expressing indicated Dar proteins of phage P1. (**B**) Protection of cells carrying pBAD_DarB or pRA plasmids and their derivatives with a premature stop codon in the beginning of *darB* (‘DarB_stop’ and ‘pRA_stop’). pRA is a plasmid encoding the first 350 N-terminal amino acids of the *darB* ORF. (**C**) Protection of cells carrying the pRA plasmid and its variants with inverted/mutated Chi site or mutated EcoKI site. (**D**) Protection of AB1157 or AB1157 *ΔclpX* cells carrying pBAD_DarB or pRA plasmids. In all panels, means from three independent experiments with standard deviations are shown.

Bacteriophage P1 Dar proteins inhibit Type I R–M defences of the *E. coli* host and deletion of the *darB* gene encoding a 2255-amino acid long protein with predicted methyltransferase and helicase domains renders the phage sensitive to EcoB and EcoK defences (44,45). To check whether the *dar* genes products are sufficient for Type I R–M inhibition, we cloned *darB*, as well as other *dar* genes and their combinations in the pBAD vector and transformed the resulting plasmids into *E. coli* AB1157 EcoKI^+^ strain. A plasmid expressing T7 Ocr, a strong inhibitor of EcoKI, was used as a control. Among various plasmids tested, only pBAD_*darB* and pBAD_*darB_ulx* made AB1157 sensitive to phage *λ* infection (Figures 1A, S1). Curiously, inactivation of the EcoK1 defence was not dependent on the presence of arabinose, an inducer of cloned genes expression (Supplementary Figure S2). Moreover, plasmids carrying *darB* variants encoding proteins with mutated predicted catalytic residues also made AB1157 sensitive to *λ* infection (data not shown). To investigate whether the anti-restriction effect is associated with the DarB protein, we introduced a premature stop codon into the full-sized plasmid-borne *darB* or into its truncated version encoding the first 350 DarB amino acids (this plasmid is hereafter referred to as pRA). Both plasmids inhibited the EcoKI defence as efficiently as the corresponding plasmids without the introduced stop codons (Figure 1B). The results thus suggest that neither the protein product of the *darB* gene nor its transcription are required for inhibition of the EcoKI defence. In other words, a fragment of *darB* gene sequence or its combination with the pBAD plasmid inhibits EcoKI.

### Chi and EcoKI sites in plasmid DNA are required to activate RA in the wild-type host background

The P1 genome is enriched with Chi sites (58); the full-sized *darB* gene carries 11 Chi sites; the *darB* fragment in the pRA plasmid has one. Other *dar* genes, which do not cause anti-restriction when present on the pBAD vector, have none. Chi sites are also not present in the pBAD sequence. The asymmetric Chi sequence (5′GCTGGTGG) is recognized by the RecBCD nuclease-helicase complex, which processes double-stranded DNA breaks (59,60). Chi sequence recognition by the RecC subunit activates the loading of RecA onto ssDNA generated by RecBCD, which promotes homologous recombination (60–62). We hypothesized that Chi-mediated plasmid recombination may be the reason for the anti-restriction activity of plasmids carrying *darB* or its fragment. Indeed, when we mutated the only Chi site present in pRA, the anti-restriction activity was lost. In contrast, reversing the orientation of Chi had no effect (Figure 1C).

The pBAD vector carries a single EcoKI recognition site in its ampicillin resistance gene *bla*. To investigate whether this EcoKI site is required for anti-restriction activity, we introduced mutations that destroyed the site without affecting the sequence of the beta-lactamase protein. Cells carrying the resulting pRA derivative were resistant to λ infection (Figure 1C). Thus, the simultaneous presence of Chi and EcoKI sites is required for plasmid-induced anti-restriction activity against EcoKI. Since the observed phenomenon is reminiscent of restriction alleviation activated by the presence of non-methylated EcoKI sites in host DNA, we tested whether plasmid-mediated anti-restriction is dependent on the ClpXP protease responsible for the HsdR subunit degradation. Anti-restriction by either pBAD_*darB* or pRA was lost in the *ΔclpX* background (Figure 1D), confirming that plasmid DNA serves as a novel input inducing ClpXP-mediated RA.

### Tn5053 anti-restriction does not depend on the *ardD* locus but is caused by plasmid-induced RA

**Figure 2.**
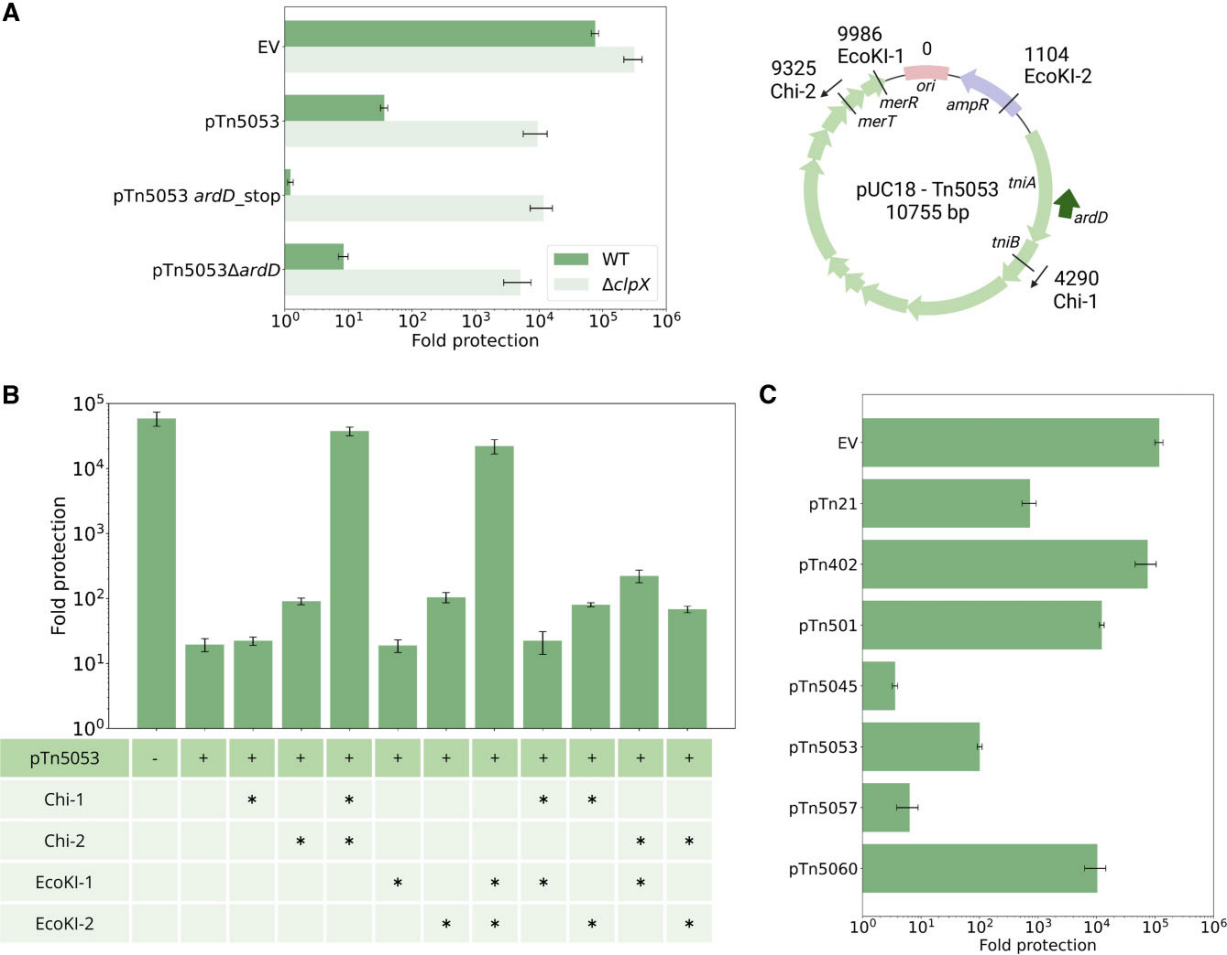
Identification of anti-restriction determinants of plasmid-encoded Tn5053. (**A**) Experiments were performed as in Figure 1 with AB1157 or AB1157*ΔclpX* cells harboring the pUC18 empty vector (‘EV’), pUC18-Tn5053, and its derivatives with a premature stop codon in, or deletion of the *ardD* gene. On the right, locations of Chi and EcoKI sites, and selected pUC18-Tn5053 genes are shown on the plasmid map. (**B**) Protection level of AB1157 cells carrying pUC18-Tn5053 and its derivatives with mutated Chi and EcoKI sites. Mutated sites are indicated by asterisks. (**C**) Protection level of AB1157 cells carrying Tn5053-related non-conjugative transposons cloned on different plasmid backbones. Levels of means from three independent experiments with standard deviations are shown.

Non-conjugative transposon Tn5053 cloned on a high-copy number pUC18 vector provides a strong anti-restriction effect in the AB1157 strain infected with *λ* phage (50). The pUC-Tn5053 induced RA is ClpXP-dependent and was linked to *ardD*, a putative protein-coding gene located within and transcribed from a different strand of the *tniA* transposase gene (49–52). The *ardD* gene expression has never been demonstrated and we did not observe any anti-restriction activity of *ardD* cloned into pBAD expression vector (Supplementary Figure S3A). We hypothesized that similarly to *darB* plasmids (above), anti-restriction associated with Tn5053 is explained by plasmid-induced RA. We introduced a premature stop-codon into the predicted *ardD* ORF without affecting the sequence of the TniA transposase and tested the resulting derivative of pUC-Tn5053 for anti-restriction. As can be seen from Figure 2A, EcoKI defence was not restored upon inactivation of the predicted *ardD* ORF. We next completely removed the *ardD* locus from pUC-Tn5053. This disrupted the *tniA* gene but had no effect on pUC-Tn5053-mediated anti-restriction (Figure 2A). We conclude, that neither *ardD* (if it exists) nor *tniA* are involved in anti-restriction by plasmid-borne Tn5053.

pUC-Tn5053 contains two Chi sites and two EcoKI sites. We inactivated each of these sites individually or in pairs and tested the resulting plasmids for the ability to alleviate EcoKI restriction. Only mutations that destroyed both EcoKI or both Chi sites inactivated anti-restriction, confirming that these effects are mediated by the DNA sequence of pUC-Tn5053 (Figure 2B). As expected, similar to *darB* fragment-induced RA, the anti-restriction activity of Tn5053 was also dependent on the presence of the ClpXP protease (Figure 2A). Since pUC-Tn5053 mediated RA was higher than that observed with pRA, it was used as the main model system in further experiments. Since inactivation of both EcoKI or Chi sites was required to inactivate RA in the wild-type background, we designate these plasmids pTn5053 EcoKI^−^ and pTn5053 Chi^−^ and use them as controls throughout the rest of this work.

To determine whether anti-restriction activity reported for non-conjugative transposons other than Tn5053 (51) is also due to plasmid-induced RA, we Illumina-sequenced plasmids with cloned transposons available to us (Supplementary Figure S3B) and determined their activity against EcoKI in the RA test (Figure 2C). All sequenced transposon-containing plasmids carried EcoKI sites. Consistent with expectations, two plasmids (carrying Tn5090 and Tn5060) that lacked Chi sites had no anti-EcoKI activity (Figure 2C, S3B). With the exception of the Tn501 plasmid, plasmids that had both EcoKI and Chi sites inhibited the EcoKI defence. The lack of activity of the Tn501 plasmid could be explained by the lower copy number of its pBR322 backbone, though pBR322 carrying Tn21 inhibited EcoKI defence (Figures 2C, S3B). Thus, additional, yet unknown plasmid features may be required to induce RA.

**Figure 3.**
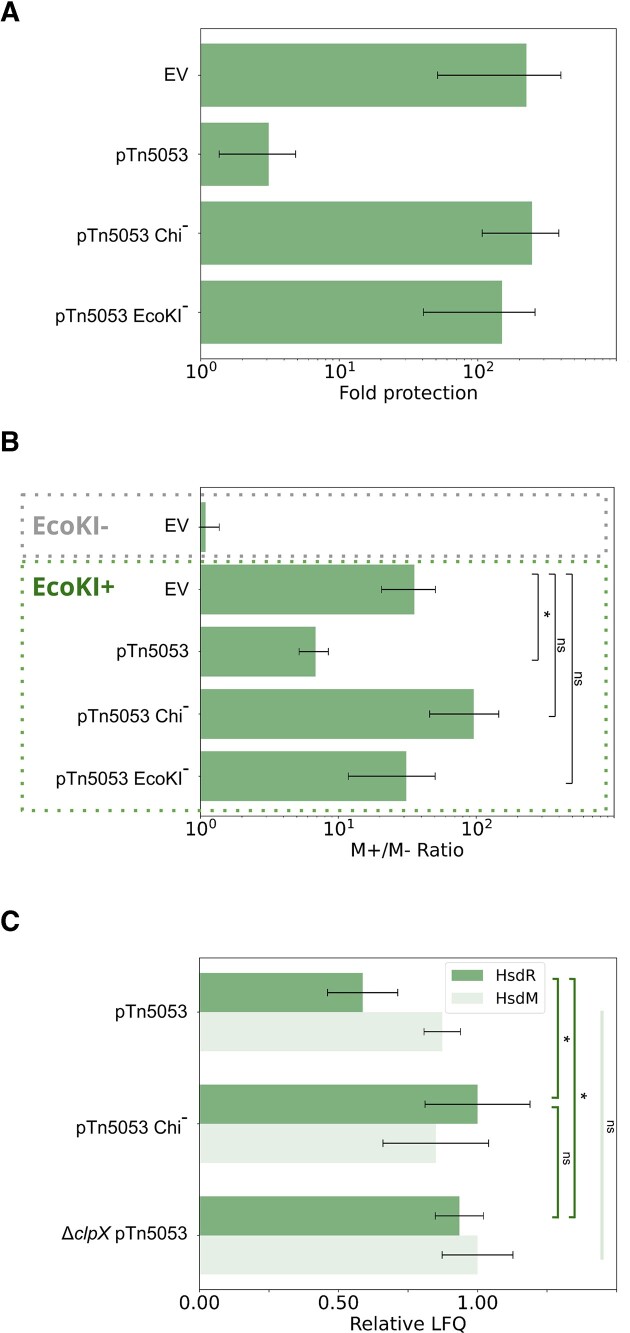
Plasmid-induced RA is not phage-specific and results in reduced levels of HsdR. (**A**) Protection against T7*Δ0.3* phage in AB1157 cells carrying indicated plasmids. Means from three independent experiments with standard deviations are shown. (**B**) Efficiency of transformation (EOT) assay with EcoKI^−^ strain (AB1157 *ΔhsdM*) and EcoKI^+^ strain AB1157 carrying pUC18-Tn5053 or its RA-inactive EcoKI^−^ and Chi^−^ derivatives. Chemocompetent cells were transformed with non-methylated or methylated variants of pTG vector (carries one EcoKI recognition site) and the results are presented as a fold-change difference in efficiency of methylated plasmid transformation relative to taht of non-methylated plasmid, thus reflecting the efficiency of EcoKI defence. Analysis was performed in biological triplicates. Statistical significance was measured using nested ANOVA coupled with one-sided *t*-test comparison using EV as reference. Adjusted p-values are reported above the graph (not significant (ns), and * *P* < 0.1). (**С**) Mass-spectrometric analysis of HsdR or HsdM subunit abundance in AB1157 cells carrying pUC18-Tn5053, or pUC18-Tn5053 variants without Chi or EcoKI sites. Analysis was performed in biological triplicates. LFQ values were normalized to the highest among three samples. Statistical significance was measured using two-way ANOVA coupled with one-sided *t*-test comparison. Adjusted *P*-values are reported above the graph (not significant (ns), and * *P* < 0.1).

To prove that plasmid-induced RA is not specific to phage *λ* proteins produced during the infection, we tested a derivative of an unrelated phage T7 for ability to overcome the EcoKI defence in the presence of pUC-Tn5053. As can be seen from Figure 3A, the anti-restriction activity of pUC-Tn5053 was readily observed with this phage.

We also tested whether pUC-Tn5053 inhibited the ability of EcoKI to decrease plasmid transformation efficiency. For this purpose, AB1157 cells bearing pUC18, pUC-Tn5053, or its derivative lacking EcoKI or Chi sites were transformed with a compatible EcoKI-methylated or non-methylated pTG plasmid carrying a single EcoKI recognition site. Cells with pUC18 control vector were transformed by non-methylated pTG ∼30 times less efficiently than by the methylated variant, as expected. The presence of pUC-Tn5053, but not its derivatives incapable of RA induction, increased the transformation efficiency of non-methylated pTG three times, confirming that plasmid-induced RA is not specific to phage infection (Figure 3B).

### Plasmid-induced RA acts through HsdR depletion and deactivates EcoKI defence in the absence of phage infection

Plasmid-mediated RA is dependent on ClpXP protease, which should inactivate the EcoKI defence through HsdR depletion. To test this prediction, we determined the levels of HsdR in AB1157 cells transformed with pUC-Tn5053. As controls we used cells transformed with pUC-Tn5053-Chi^−^ plasmids that lack Chi sites and do not affect the EcoKI activity (above). AB1157 *ΔclpX* cells carrying pUC-Tn5053 served as another control. Cell lysates were subjected to mass-spectrometric proteomic analysis and the HsdR signal was compared with the signal from the HsdM subunit, which should not be affected by ClpXP proteolysis (30). According to LFQ analysis, HsdR was ∼2 times less abundant in cells with pUC-Tn5053 compared to cells with pUC-Tn5053 bearing mutated Chi sites or cells with deleted *clpX* gene (Figure 3C). The result is thus consistent with an expectation that in the presence of an RA-capable plasmid the intracellular amounts of HsdR are reduced due to ClpXP-mediated proteolysis.

### Plasmid-induced RA can proceed via Chi- and RecBCD-independent pathway

**Figure 4.**
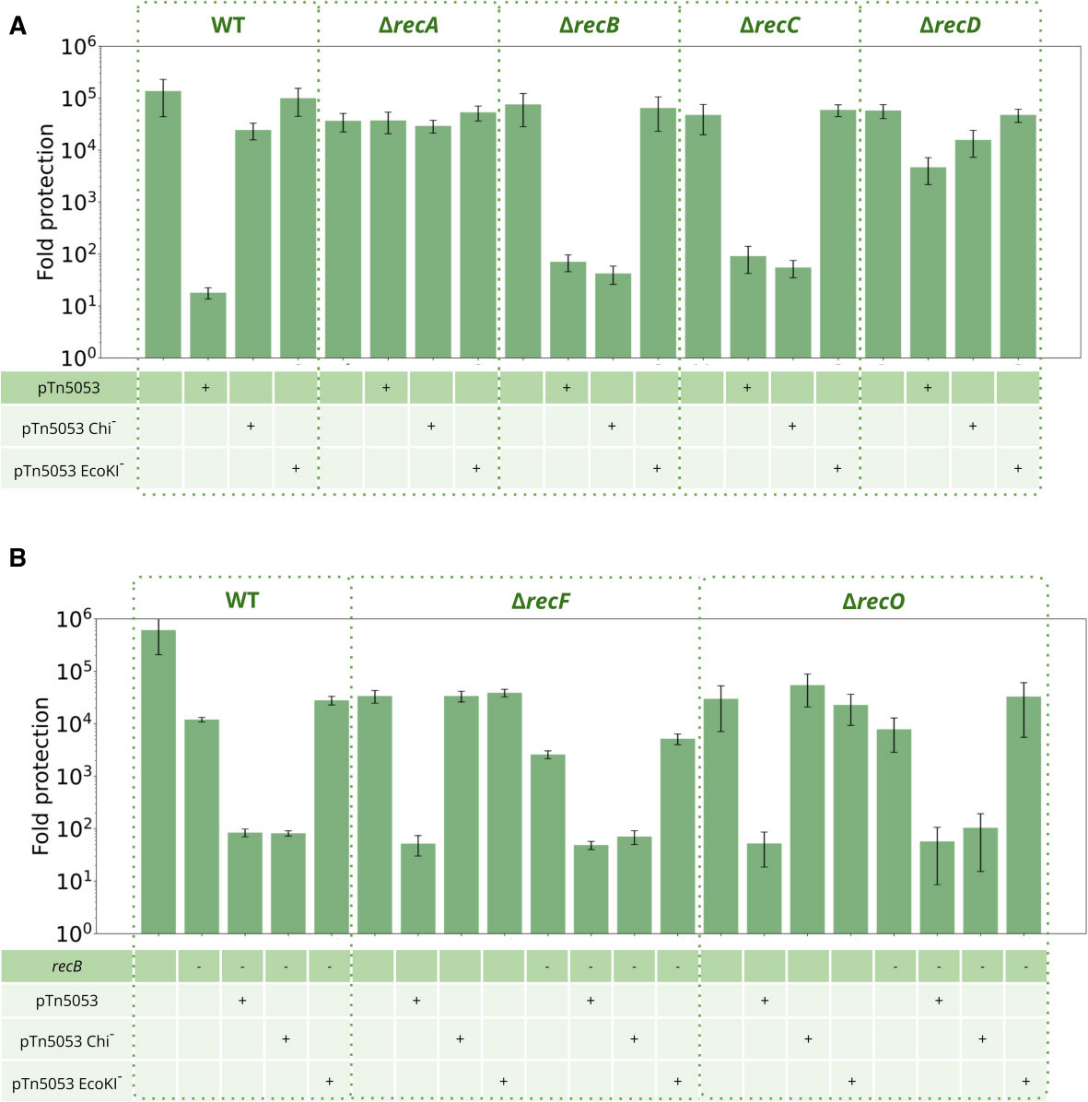
Chi-sites carrying plasmids require the presence of RecA and RecBCD for RA induction; RecBC inactivation relieves the Chi site requirement. (**A**) Levels of protection against *λ_vir_* infection of cells carrying pUC18-Tn5053 and its variants with mutated Chi and EcoKI sites. The AB1157 strains genetic backgrounds are indicated at the top. (**B**) Double deletions of *recB* with *recF* or *recO* do not affect RA. Experiment was performed as in panel A. Means from three independent experiments with standard deviations are shown.

Since plasmid-induced RA was dependent on the presence of at least one Chi site, we tested the involvement of RecA and RecBCD proteins using appropriate mutants. The *λ_vir_* titers were the same on EcoKI^−^ AB1157*ΔhsdM rec^+^* cell lawns and on lawns formed by the isogenic *rec* mutants, indicating that mutations do not affect phage growth. Each strain was transformed with pUC-Tn5053 or EcoKI^−^/Chi^−^ derivative plasmids and infected with *λ_vir_*(Figure 4A). pUC-Tn5053 EcoKI^−^ was inactive in all backgrounds, confirming the strict requirement for at least one EcoKI site for RA. The *recA* deletion abolished pUC-Tn5053-induced RA, suggesting that RecA-mediated homologous recombination is required for RA. However, deletions of genes coding for the RecB and RecC subunits, which are essential for the activity of the RecBCD complex, still permitted RA with pTn5053. The result suggests that RA is not strictly dependent on RecBCD and Chi sites recognition mediated by RecC (63). Accordingly, Chi-lacking pTn5053 was anti-restriction active in the *recB* or *recC* deletion backgrounds (Figure 4A). Why then the Chi-deficient pTn5053 did not induce RA in the wild-type strain? We hypothesize that in the absence of RecBCD processing, Chi-lacking plasmids that sustain double-stranded breaks still undergo recombination that generates non-methylated EcoKI sites, while in the presence of RecBCD, linearized Chi-deficient plasmids are completely degraded. Thus, alternative RecA-dependent or RecA-independent recombination pathways manifest themselves only in the absence of RecBCD processing. The major alternative RecA-loading recombination pathway is mediated by the RecFOR proteins (64,65). Double *recF recB* or *recO recB* mutants alleviated restriction when transformed with pUC-Tn5053, suggesting the existence of pathway(s) supporting plasmid recombination in the absence of either RecBCD or RecFOR (Figure 4B).

The *recD* deletion also abolished RA (Figure 4A). The RecBC complex lacking RecD has significantly reduced exonuclease/helicase activity but retains the ability to bind dsDNA substrates and promotes Chi-independent recombination (66,67). We speculate that at conditions of decreased dsDNA nucleolytic processing recombination of damaged plasmid DNA does not efficiently generate non-methylated EcoKI sites, while the binding of the RecBC complex still blocks alternative recombination pathways active in the *ΔrecB/recC* background.

### Plasmid-induced RA can be activated through RecA-independent pathway

**Figure 5.**
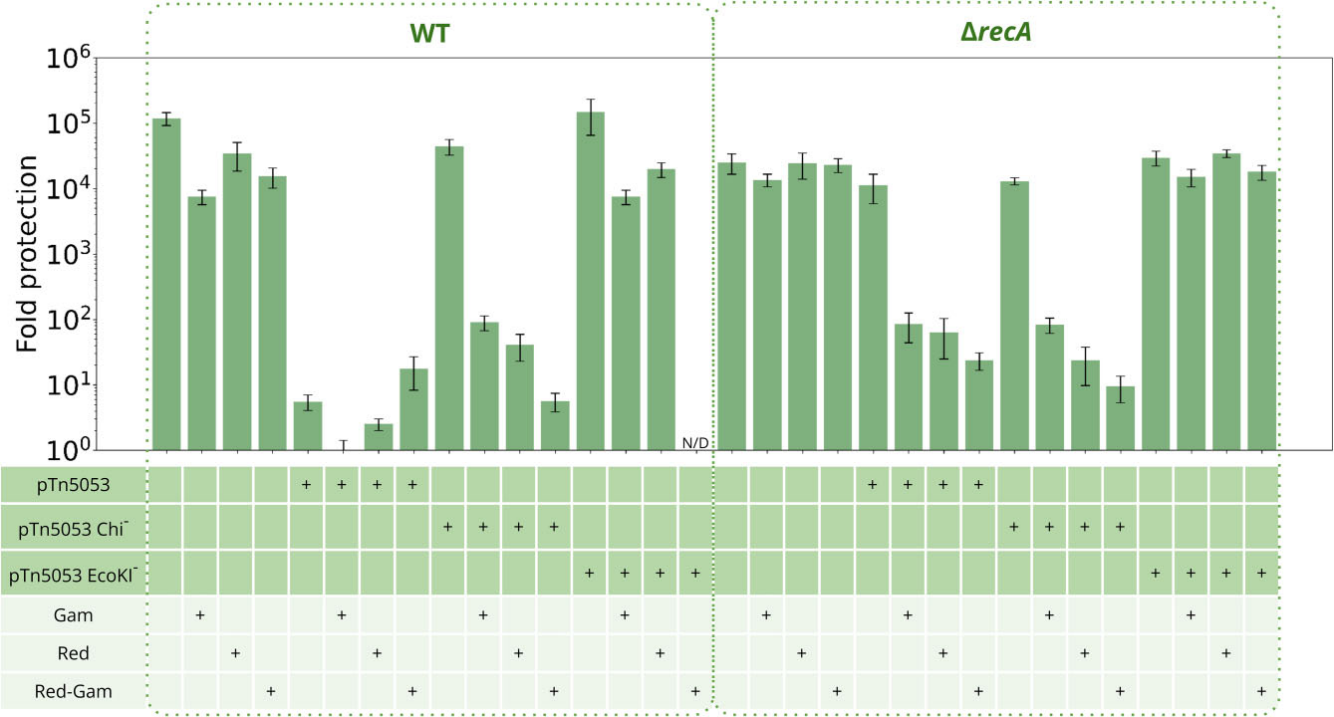
RecBCD inhibitor *λ* Gam or *λ* Red homologous recombination system induce Chi-independent plasmid-mediated RA. Levels of protection from *λ_vir_* infection for cells carrying pUC18-Tn5053 and its variants with mutated Chi and EcoKI sites. AB1157 strains genetic backgrounds are indicated at the top. Cells were grown in the presence of 0.2% l-arabinose to induce *λ* proteins synthesis. N/D, not determined.

If recombination of Chi-lacking plasmids in the *ΔrecA* background is blocked due to their RecBCD-mediated degradation, both RecA and RecBCD should be eliminated to investigate the contribution of RecA-independent pathways. Despite multiple attempts, we were unable to obtain a *ΔrecAB* deletion using P1 transduction. We therefore inactivated RecBCD in the *ΔrecA* background by expressing the RecBCD inhibitor—a DNA mimicking phage *λ* protein Gam. We also investigated the ability of an alternative recombination system *λ* Red to promote Chi- and RecA-independent recombination and induce RA. For this purpose, *λ* Gam, *λ* Red, or *λ* Red *and* Gam were overproduced from a compatible vector in the presence of pUC-Tn5053 or its EcoKI^−^/Chi^−^ derivatives in the wild-type or *ΔrecA* AB1157 strains (Figure 5). Though *λ* Gam was previously reported to affect the EcoKI defence (68), no effects of *λ* proteins on the EcoKI defence was observed in our system. The pUC-Tn5053 EcoKI^−^ plasmid had no RA activity at all conditions tested, confirming the strict requirement for the EcoKI restriction complex interaction with plasmid DNA for RA induction. While the pUC-Tn5053 Chi^−^ plasmid had no RA activity in cells with active RecABCD, inhibition of RecBCD by Gam or the presence of Chi-independent *λ* Red recombination system made this plasmid RA-proficient. We suppose that even in the absence of Gam, the λ RED system overexpressed from an inducible promoter outcompetes RecBCD for binding to dsDNA ends, thus promoting RecA-independent recombination. This recapitulates the result obtained in the *ΔrecB* background and confirms a model according to which plasmid-mediated RA requires ongoing recombination. Finally, while both pUC-Tn5053 and pUC-Tn5053 Chi^−^ plasmids were inactive on the *ΔrecA* background, expression of *λ* Gam restored RA in this strain, further confirming that RecA-independent pathways can mediate plasmid recombination. The activity of these pathways becomes evident only when RecBCD is inactivated.

### Chi-site bearing plasmids activate the SOS response, yet RA is not a SOS response function

Chi-dependent generation of non-methylated sites through recombination requires dsDNA breaks that attract the RecBCD complex. Processing of dsDNA breaks by RecBCD leads to RecA-mediated induction of the SOS response (69–72). To investigate if RA-inducing plasmids activate the SOS response, we used the MP062 *E. coli* strain with a Yellow Fluorescent Protein (YFP) gene cloned under the control of the SOS-responsive *sulA* promoter (5). Treatment of MP062 cells with DNA damaging nalidixic acid resulted in accumulation of the YFP signal, indicating the activation of the SOS response. The presence of pUC-Tn5053 and pUC-Tn5053 EcoKI^−^, but not of pUC-Tn5053 Chi^−^ or the pUC18 control, also resulted in slow accumulation of the YFP signal at later time points (Supplementary Figure S4A). None of the plasmids induced the SOS response in the MP074 strain, an MP062 derivative deficient in *recA*. We conclude that low-level SOS response is activated through accumulation of dsDNA breaks in high-copy number plasmids carrying Chi-sites. To further confirm this observation, we investigated the SOS-response signal from individual cells using flow cytometry. We determined the proportion of SOS-activated MP062 cells treated with a sublethal concentration of nalidixic acid (2.5 μg/ml) or grown in the presence of pUC18, pUC-Tn5053, or EcoKI^−^/Chi^−^ derivatives (Supplementary Figure S4B). Cells were gated at 5% of the highest YFP signal in the pUC18 control. ∼30% of cells treated with nalidixic acid were above this threshold. While pTn5053 Chi^−^ culture was close to the pUC control (∼5.7%), the proportion of pTn5053 and pTn5053 EcoKI^−^ cells showing signal above the threshold was higher (∼7% and 8%, correspondingly), suggesting weak activation of the SOS response in a subpopulation of cells carrying plasmids with a Chi-site.

In early works, RA was considered as one of the outcomes of the SOS-response (73). To exclude a possibility that RA is dependent on SOS activation, we tested AB1157-derived *lexA1* strain AB2494 (74). The SOS response relies on the interaction of activated RecA and transcriptional repressor LexA, which induces LexA autoproteolysis and derepresses the SOS regulon (75–77). LexA1 is defective in autoproteolysis and remains bound to SOS-activated promoters even in the presence of activated RecA (78). In the *lexA1* mutant background pUC-Tn5053 retained full anti-restriction activity, suggesting that SOS response activation is not required for EcoKI defence inhibition (Supplementary Figure S5). Thus, we conclude that even though pUC-Tn5053 activates SOS response in a sub-population of cells, plasmid-mediated RA does not depend on the SOS response activation.

## Discussion

**Figure 6.**
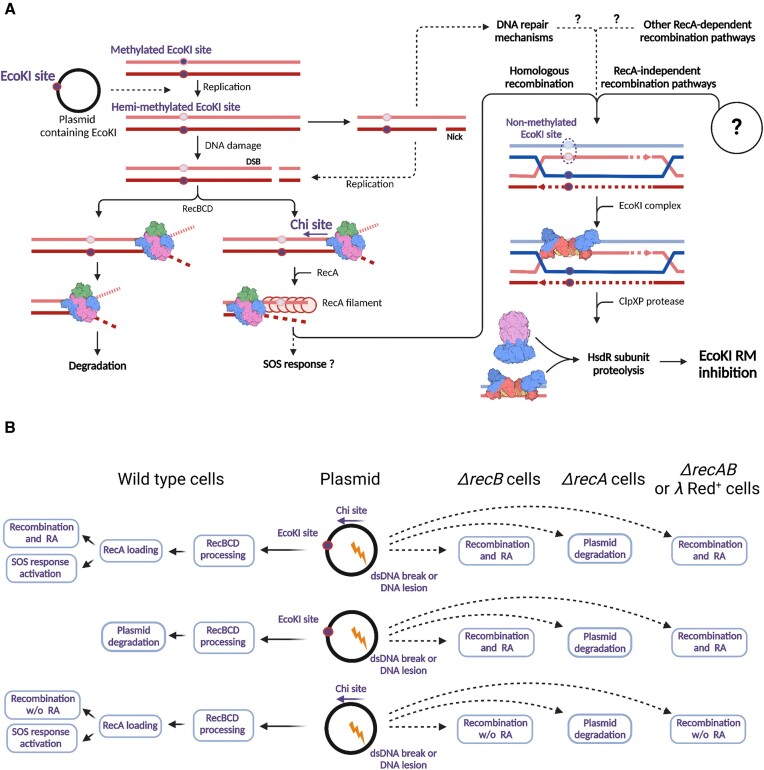
A model of plasmid-induced RA. (**A**) Proposed sequence of events leading to the generation of non-methylated EcoKI sites in plasmid DNA and the launch of RA. See text for details. (**B**) The presence of plasmids activates RA, induces SOS response or produces no specific phenotype, depending on the host background and the presence of Chi and EcoKI sites in the plasmid DNA.

In this work, we demonstrate that plasmids can alleviate EcoKI restriction. A model of plasmid-induced RA is presented in Figure 6A and is supported by multiple lines of evidence. We envision that a cascade of events resulting in the EcoKI defence inhibition begins with accumulation of spontaneous double-stranded breaks in plasmid DNA. Free DNA ends attract RecBCD, which completely degrades linearized plasmids lacking Chi sites (62). If a plasmid processed by RecBCD contains at least one Chi site, the RecA-dependent homologous recombination is activated (79). Recombination between homologous regions containing hemi-methylated EcoKI sites that transiently accumulate each time plasmid DNA is replicated generates non-methylated sites. Binding of EcoKI restriction complexes to non-methylated sites activates DNA translocation, which exposes the HsdR subunit to ClpXP proteolysis. Thus, plasmid DNA serves as a ‘sponge’ that constantly attracts the EcoKI restriction complexes and channels them for proteolytic degradation. This process happens only in conditions of active plasmid recombination that generates non-methylated EcoKI sites. The same recombination process should take place in the *E. coli* chromosomal DNA, yet in previous works RA was observed only at conditions of increased DNA damage (25,27). Presumably, the frequency of generation of non-methylated EcoKI sites in plasmid but not host DNA is high enough at normal growth conditions to produce RA in a non-damaged cell. It should be noted that the HsdR levels are not increased in *ΔclpX* strains, confirming that in the lack of additional inputs RA is not induced by the chromosomal DNA (29). The difference is likely due to the higher copy number of plasmid DNA, which should increase the frequency of recombination.

Our model explains several previously observed phenomena of EcoKI defence inhibition. First, it was reported that the EcoKI defence is reduced in *recB-*deficient cells carrying the pACYC184 plasmid (68). pACYC184 carries one EcoKI site but lacks Chi sequences and thus is similar to pUC Tn5053 Chi^−^, which in our work was active only in *recB*-deficient cells. Presumably, in the wild-type cells random DSBs sustained by pACYC184 or Tn5053 Chi^−^ plasmids lead to degradation by RecBCD, while the *recB* deletion allows recombination or repair to proceed via Chi-independent ways, leading to the launch of RA. The second phenomenon concerns the proposed anti-restriction gene *ardD* of non-conjugative transposons (50). We show that *ardD* does not contribute to anti-restriction, which is mediated by the plasmid DNA itself. Whether *ardD*, one of the rare examples of a gene encoded in an anti-sense orientation within another gene (*tniA*), is real remains to be shown, but it clearly has no role in RA. In fact, our results show that caution should be taken during studies of anti-restriction mechanisms acting against Type I RM systems, such as, for example, DarB, since plasmid- and protein-mediated anti-restriction mechanism can be separated only in conditions of blocked RA (a *ΔclpX* background or expression from plasmids lacking RM recognition sites).

In the wild-type cells, plasmid-induced RA is dependent on RecA and RecBCD (Figures 1 and 2), the components of the dominant homologous recombination pathway in *E. coli*. Deletion of *recA* in the presence of RecBCD completely abolishes RA (Figure 4A). However, inactivation of RecB or RecC relieves the Chi site requirement for RA, implying the existence of RecBCD-independent mechanism(s) that mediate plasmid recombination and activate RA. RecA can be loaded on single-stranded DNA by the RecFOR proteins (64,80). Yet, double inactivation of *recB* and either *recF* or *recO* did not affected RA, suggesting the existence of additional RecA-loading pathways. Alternatively, RecA-independent pathways could be activated in the *recB* deletion background. We hypothesized that the lack of recombination in *ΔrecA* cells could be explained by the RecBCD-mediated degradation of linearized plasmid DNA (62). In this scenario, simultaneous inactivation of RecA and RecBCD should allow RecA-independent plasmid recombination to proceed. Indeed, expression of RecBCD inhibitor *λ* Gam restored plasmid-induced RA in the *ΔrecA* background. RecA-independent recombination in *E. coli* was reported in multiple works (81–85), but in contrast to the RecA-mediated pathway, its mechanisms are much less understood, and many proteins (e.g. RarA, RuvABC, RecJ, RecQ, UvrD, RadA, etc.) may be involved (65). In addition to homologous recombination, DNA lesions can be processed via alternative pathways, such as fork reversal, template switching or translesion DNA synthesis. Some of these pathways can be launched by the presence of DNA nicks and thus do not require free DNA ends (65). We cannot exclude that in the lack of RecBCD and/or RecA, these recombinational repair pathways also generate non-methylated EcoKI sites in plasmid DNA, however, the strict requirements for the Chi site in the wild-type background questions this hypothesis. Additional studies are required to determine *E. coli* genes involved in plasmid recombination in different host backgrounds. Thus, generation of non-methylated EcoKI sites in plasmid DNA can proceed via multiple pathways, which could be RecBCD-, RecA- and Chi-dependent or independent, and depending on the presence of active RecBCD dsDNA breaks processing could result in various outcomes, such as complete degradation of plasmid DNA, repair or homologous recombination leading to RA activation (Figure 6B).

It was shown before that conjugative plasmids can induce SOS response during conjugative transfer, when single-stranded plasmid DNA entering the cell attracts RecA (86). Thus, many conjugative plasmids express SOS response inhibitors from early single-strand specific promoters (87,88). In addition, some plasmids trigger SOS response due to expression of toxic proteins or sequestration of host replication machinery components (89–92). We here show that the presence of a Chi-bearing plasmid DNA is sufficient to induce SOS response (Supplementary Figure S4). Chi sites are ∼6.7 times overrepresented compared to other octonucleotide sequences in the *E. coli* genome and some plasmids and phages, for example, P1, show even more dramatic accumulation of Chi sequences (93). It was proposed that clashes between replication and transcription machinery could decrease plasmid stability (94). Chi-mediated recombination could rescue plasmids that suffer dsDNA breakage events and would otherwise be destroyed by RecBCD. Thus, Chi sites could be beneficial for plasmids. Given that Chi sites recognition also launches SOS response, maintenance of extrachromosomal mobile elements containing these sites could increase host mutation rates and promote acquisition of antibiotic resistance (95,96).

It should be noted that the RA phenomenon discussed in this work is mediated by plasmid DNA present inside the cell, which, therefore, must be considered as self. However, a question remains whether RA can be activated in natural settings by non-self plasmid or phage DNA upon entry into the cell. We tested whether the presence of a Chi site could represent a novel HGT-enhancing strategy and investigated the rates of pUC-Tn5053 *de novo* acquisition by EcoKI^+^ cells. Chi^+^ and Chi^−^ non-methylated plasmids were transformed with a similarly reduced efficiency compared to cognate methylated variants, demonstrating that a RA-competent plasmid does not increase the rate of its own transformation into EcoKI^+^ host (Supplementary Figure S6). In fact, once a plasmid is established within a cell, activation of RA could have negative effects by making the host (and, therefore, the plasmid it carries) susceptible to other mobile genetic elements, including phages. Thus, plasmid-induced RA is unlikely to have evolved as a dedicated way to downregulate host R–M systems and instead may represent an example of a conflict between host immunity systems and recently (in evolutionary time scales) acquired mobile elements. The principal difference between the self and non-self DNA is in the density of non-methylated sites, and it is speculated that RA is not activated during phage infection, since proteolysis-competent restriction complexes are short-lived on a DNA densely populated with non-methylated sites (30). However, it remains possible that mobile elements with low density of recognition sites will trigger RA, and although this will not increase the efficiency of colonisation of their hosts, removal of HsdR could favor co-infection by EcoKI-sensitive phages. It remains to be established whether such complex scenarios are possible, how widespread is the RA-like control in other phage defence systems, and whether similar conflicts between mobile elements and immunity systems are common in nature.

## Supplementary Material

gkae243_Supplemental_File

## Data Availability

The data underlying this article are available in the article and in its online Supplementary data. All information is available upon request to the lead contact—Dr Artem Isaev. Sequencing data have been deposited in NCBI database with accession number PRJNA1019354. Mass-spectrometry raw spectra have been deposited to PRIDE database with accession number PXD050621.

## References

[1] Hampton H.G., Watson B.N.J., Fineran P.C. The arms race between bacteria and their phage foes. Nature. 2020; 577:327–336. 31942051 10.1038/s41586-019-1894-8

[2] Samson J.E., Magadán A.H., Sabri M., Moineau S. Revenge of the phages: defeating bacterial defences. Nat. Rev. Micro. 2013; 11:675–687. 10.1038/nrmicro3096 23979432

[3] Mayo-Muñoz D., Pinilla-Redondo R., Birkholz N., Fineran P.C. A host of armor: Prokaryotic immune strategies against mobile genetic elements. Cell Rep. 2023; 42:112672. 37347666 10.1016/j.celrep.2023.112672

[4] Stern A., Keren L., Wurtzel O., Amitai G., Sorek R. Self-targeting by CRISPR: gene regulation or autoimmunity?. Trends Genet. 2010; 26:335–340. 20598393 10.1016/j.tig.2010.05.008 PMC2910793

[5] Pleška M., Qian L., Okura R., Bergmiller T., Wakamoto Y., Kussell E., Guet C.C. Bacterial autoimmunity due to a restriction–modification system. Curr. Biol. 2016; 26:404–409. 26804559 10.1016/j.cub.2015.12.041

[6] Semenova E., Minakhin L., Bogdanova E., Nagornykh M., Vasilov A., Heyduk T., Solonin A., Zakharova M., Severinov K. Transcription regulation of the EcoRV restriction–modification system. Nucleic Acids Res. 2005; 33:6942–6951. 16332697 10.1093/nar/gki998 PMC1310966

[7] Mruk I., Kobayashi I. To be or not to be: regulation of restriction–modification systems and other toxin–antitoxin systems. Nucleic Acids Res. 2014; 42:70–86. 23945938 10.1093/nar/gkt711 PMC3874152

[8] Keller L.M.L., Weber-Ban E. An emerging class of nucleic acid-sensing regulators in bacteria: WYL domain-containing proteins. Curr. Opin. Microbiol. 2023; 74:102296. 37027901 10.1016/j.mib.2023.102296

[9] Høyland-Kroghsbo N.M., Paczkowski J., Mukherjee S., Broniewski J., Westra E., Bondy-Denomy J., Bassler B.L. Quorum sensing controls the Pseudomonas aeruginosa CRISPR-Cas adaptive immune system. Proc. Natl. Acad. Sci. U.S.A. 2017; 114:131–135. 27849583 10.1073/pnas.1617415113 PMC5224376

[10] Patterson A.G., Jackson S.A., Taylor C., Evans G.B., Salmond G.P.C., Przybilski R., Staals R.H.J., Fineran P.C. Quorum sensing controls adaptive immunity through the regulation of multiple CRISPR-Cas systems. Mol. Cell. 2016; 64:1102–1108. 27867010 10.1016/j.molcel.2016.11.012 PMC5179492

[11] Athukoralage J.S., White M.F. Cyclic oligoadenylate signaling and regulation by ring nucleases during type III CRISPR defense. RNA. 2021; 27:855–867. 33986148 10.1261/rna.078739.121 PMC8284326

[12] Ye Q., Lau R.K., Mathews I.T., Birkholz E.A., Watrous J.D., Azimi C.S., Pogliano J., Jain M., Corbett K.D. HORMA domain proteins and a Trip13-like ATPase regulate bacterial cGAS-like enzymes to mediate bacteriophage immunity. Mol. Cell. 2020; 77:709–722. 31932165 10.1016/j.molcel.2019.12.009 PMC7036143

[13] Bernheim A., Sorek R. The pan-immune system of bacteria: antiviral defence as a community resource. Nat. Rev. Micro. 2020; 18:113–119. 10.1038/s41579-019-0278-2 31695182

[14] Ipoutcha T., Tsarmpopoulos I., Gourgues G., Baby V., Dubos P., Hill G.E., Dowling A., Arfi Y., Lartigue C., Thebault P. Evolution of the CRISPR-Cas9 defence system following a bacterial host shift. 2023; bioRxiv doi:14 March 2023, preprint: not peer reviewed 10.1101/2023.03.14.532377. PMC11893278 39556419

[15] Hussain F.A., Dubert J., Elsherbini J., Murphy M., Vaninsberghe D., Arevalo P., Kauffman K., Rodino-Janeiro B.K., Gavin H., Gomez A. Rapid evolutionary turnover of mobile genetic elements drives bacterial resistance to phages. Science. 2021; 374:488–492. 34672730 10.1126/science.abb1083

[16] Piel D., Bruto M., Labreuche Y., Blanquart F., Goudenège D., Barcia-Cruz R., Chenivesse S., Le Panse S., James A., Dubert J. Phage–host coevolution in natural populations. Nat. Microbiol. 2022; 7:1075–1086. 35760840 10.1038/s41564-022-01157-1

[17] Murray N.E. Type I restriction systems: sophisticated molecular machines (a legacy of Bertani and Weigle). Microbiol. Mol. Biol. Rev. 2000; 64:412–434. 10839821 10.1128/mmbr.64.2.412-434.2000 PMC98998

[18] Gao Y., Cao D., Zhu J., Feng H., Luo X., Liu S., Yan X.-X., Zhang X., Gao P. Structural insights into assembly, operation and inhibition of a type I restriction–modification system. Nat. Microbiol. 2020; 5:1107–1118. 32483229 10.1038/s41564-020-0731-z

[19] Neaves K.J., Cooper L.P., White J.H., Carnally S.M., Dryden D.T.F., Edwardson J.M., Henderson R.M. Atomic force microscopy of the EcoKI Type I DNA restriction enzyme bound to DNA shows enzyme dimerization and DNA looping. Nucleic Acids Res. 2009; 37:2053–2063. 19223329 10.1093/nar/gkp042 PMC2665228

[20] Ellis D.J., Dryden D.T.F., Berge T., Edwardson J.M., Henderson R.M. Direct observation of DNA translocation and cleavage by the EcoKI endonuclease using atomic force microscopy. Nat. Struct. Biol. 1999; 6:15–17. 9886284 10.1038/4882

[21] Loenen W.A.M. Tracking EcoKI and DNA fifty years on: A golden story full of surprises. Nucleic Acids Res. 2003; 31:7059–7069. 14654681 10.1093/nar/gkg944 PMC291878

[22] Ishikawa K., Handa N., Kobayashi I. Cleavage of a model DNA replication fork by a Type I restriction endonuclease. Nucleic Acids Res. 2009; 37:3531–3544. 19357093 10.1093/nar/gkp214 PMC2699502

[23] Murray N.E. Immigration control of DNA in bacteria: self versus non-self. Microbiology (N Y). 2002; 148:3–20. 10.1099/00221287-148-1-3 11782494

[24] Bertani G., Weigle J.J. Host controlled variation in bacterial viruses. J. Bacteriol. 1953; 65:113. 13034700 10.1128/jb.65.2.113-121.1953 PMC169650

[25] Day R.S. 3rd UV-induced alleviation of K-specific restriction of bacteriophage lambda. J. Virol. 1977; 21:1249–1251. 321803 10.1128/jvi.21.3.1249-1251.1977 PMC515672

[26] Thoms B., Wackernagel W. UV-induced allevation of λ restriction in Escherichia coli K-12: kinetics of induction and specificity of this SOS function. Mol. Gen. Genet. 1982; 186:111–117. 6213835 10.1007/BF00422921

[27] Efimova E.P., Delver E.P., Belogurov A.A. 2-Aminopurine and 5-bromouracil induce alleviation of type I restriction in Escherichia coli: mismatches function as inducing signals?. Mol. Gen. Genet. 1988; 214:317–320. 2976882 10.1007/BF00337728

[28] Makovets S., Titheradge A.J.B., Murray N.E. ClpX and ClpP are essential for the efficient acquisition of genes specifying type IA and IB restriction systems. Mol. Microbiol. 1998; 28:25–35. 9593294 10.1046/j.1365-2958.1998.00767.x

[29] Makovets S., Doronina V.A., Murray N.E. Regulation of endonuclease activity by proteolysis prevents breakage of unmodified bacterial chromosomes by type I restriction enzymes. Proc. Natl. Acad. Sci. U.S.A. 1999; 96:9757–9762. 10449767 10.1073/pnas.96.17.9757 PMC22283

[30] Simons M., Diffin F.M., Szczelkun M.D. ClpXP protease targets long-lived DNA translocation states of a helicase-like motor to cause restriction alleviation. Nucleic Acids Res. 2014; 42:12082–12091. 25260590 10.1093/nar/gku851 PMC4231737

[31] Doronina V.A., Murray N.E. The proteolytic control of restriction activity in Escherichia coli K-12. Mol. Microbiol. 2001; 39:416–429. 11136462 10.1046/j.1365-2958.2001.02232.x

[32] Keatch S.A., Su T.-J., Dryden D.T.F. Alleviation of restriction by DNA condensation and non-specific DNA binding ligands. Nucleic Acids Res. 2004; 32:5841–5850. 15520467 10.1093/nar/gkh918 PMC528803

[33] Blakely G.W., Murray N.E. Control of the endonuclease activity of type I restriction–modification systems is required to maintain chromosome integrity following homologous recombination. Mol. Microbiol. 2006; 60:883–893. 16677300 10.1111/j.1365-2958.2006.05144.x

[34] Makovets S., Powell L.M., Titheradge A.J.B., Blakely G.W., Murray N.E. Is modification sufficient to protect a bacterial chromosome from a resident restriction endonuclease?. Mol. Microbiol. 2004; 51:135–147. 14651617 10.1046/j.1365-2958.2003.03801.x

[35] Seidel R., Bloom J.G.P., van Noort J., Dutta C.F., Dekker N.H., Firman K., Szczelkun M.D., Dekker C. Dynamics of initiation, termination and reinitiation of DNA translocation by the motor protein Eco R124I. EMBO J. 2005; 24:4188–4197. 16292342 10.1038/sj.emboj.7600881 PMC1356320

[36] Rusinov I.S., Ershova A.S., Karyagina A.S., Spirin S.A., Alexeevski A.V. Avoidance of recognition sites of restriction–modification systems is a widespread but not universal anti-restriction strategy of prokaryotic viruses. Bmc Genomics [Electronic Resource]. 2018; 19:885. 30526500 10.1186/s12864-018-5324-3 PMC6286503

[37] Shaw L.P., Rocha E.P.C., MacLean R.C. Restriction-modification systems have shaped the evolution and distribution of plasmids across bacteria. Nucleic Acids Res. 2023; 51:6806–6818. 37254807 10.1093/nar/gkad452 PMC10359461

[38] Weigele P., Raleigh E.A. Biosynthesis and function of modified bases in bacteria and their viruses. Chem. Rev. 2016; 116:12655–12687. 27319741 10.1021/acs.chemrev.6b00114

[39] Atanasiu C., Su T.J., Sturrock S.S., Dryden D.T.F. Interaction of the ocr gene 0.3 protein of bacteriophage T7 with EcoKl restriction/modification enzyme. Nucleic Acids Res. 2002; 30:3936–3944. 12235377 10.1093/nar/gkf518 PMC137103

[40] Spoerel N., Herrlich P. Colivirus-T3-coded S-adenosylmethionine hydrolase. Eur. J. Biochem. 1979; 95:227–233. 110588 10.1111/j.1432-1033.1979.tb12957.x

[41] Guo X., Söderholm A., Isaksen G.V., Warsi O., Eckhard U., Trigüis S., Gogoll A., Jerlström-Hultqvist J., Åqvist J., Andersson D.I. Structure and mechanism of a phage-encoded SAM lyase revises catalytic function of enzyme family. eLife. 2021; 10:e61818. 33567250 10.7554/eLife.61818 PMC7877911

[42] Isaev A., Drobiazko A., Sierro N., Gordeeva J., Yosef I., Qimron U., Ivanov N.V., Severinov K Phage T7 DNA mimic protein Ocr is a potent inhibitor of BREX defence. Nucleic Acids Res. 2020; 48:5397–5406. 32338761 10.1093/nar/gkaa290 PMC7261183

[43] Andriianov A., Trigüis S., Drobiazko A., Sierro N., Ivanov N.V., Selmer M., Severinov K., Isaev A. Phage T3 overcomes the BREX defense through SAM cleavage and inhibition of SAM synthesis by SAM lyase. Cell Rep. 2023; 42:112972. 37578860 10.1016/j.celrep.2023.112972

[44] Iida S., Streiff M.B., Bickle T.A., Arber W. Two DNA antirestriction systems of bacteriophage P1, darA, and darB: characterization of darA− phages. Virology. 1987; 157:156–166. 3029954 10.1016/0042-6822(87)90324-2

[45] Piya D., Vara L., Russell W.K., Young R., Gill J.J. The multicomponent antirestriction system of phage P1 is linked to capsid morphogenesis. Mol. Microbiol. 2017; 105:399–412. 28509398 10.1111/mmi.13705 PMC6011833

[46] McMahon S.A., Roberts G.A., Johnson K.A., Cooper L.P., Liu H., White J.H., Carter L.G., Sanghvi B., Oke M., Walkinshaw M.D. Extensive DNA mimicry by the ArdA anti-restriction protein and its role in the spread of antibiotic resistance. Nucleic Acids Res. 2009; 37:4887–4897. 19506028 10.1093/nar/gkp478 PMC2731889

[47] Serfiotis-Mitsa D., Herbert A.P., Roberts G.A., Soares D.C., White J.H., Blakely G.W., Uhrín D., Dryden D.T.F. The structure of the KlcA and ArdB proteins reveals a novel fold and antirestriction activity against Type I DNA restriction systems in vivo but not in vitro. Nucleic Acids Res. 2010; 38:1723–1737. 20007596 10.1093/nar/gkp1144 PMC2836571

[48] González-Montes L., Del Campo I., Garcillán-Barcia M.P., de la Cruz F., Moncalián G. ArdC, a ssDNA-binding protein with a metalloprotease domain, overpasses the recipient hsdRMS restriction system broadening conjugation host range. PLoS Genet. 2020; 16:e1008750. 32348296 10.1371/journal.pgen.1008750 PMC7213743

[49] Rastorguev S.M., Letuchaia T.A., GIa K., Mindlin S.Z., Nikiforov V.G., Zavil’gel'skii˘ G.B. Antirestriction activity of metalloregulatory proteins ArsR and MerR. Mol. Biol. (Mosk.). 1999; 33:203–206. 10377563

[50] Balabanov V.P., Kotova V.Y., Kholodii G.Y., Mindlin S.Z., Zavilgelsky G.B. A novel gene, ardD, determines antirestriction activity of the non-conjugative transposon Tn 5053 and is located antisense within the tniA gene. FEMS Microbiol. Lett. 2012; 337:55–60. 22967207 10.1111/1574-6968.12005 PMC3533173

[51] Zavilgelsky G.B., Kotova V.Y., Melkina O.E., Balabanov V.P., Mindlin S.Z. Proteolytic control of the antirestriction activity of Tn 21, Tn 5053, Tn 5045, Tn 501, and Tn 402 non-conjugative transposons. Mol. Biol. 2015; 49:295–302. 26065261

[52] Zavilgelsky G.B., Kotova V.Y., Melkina O.E., Pustovoit K.S. Antirestriction activity of the mercury resistance nonconjugative transposon Tn 5053 is controlled by the protease ClpXP. Russ. J. Genet. 2014; 50:910–915. 25735133

[53] Lee D.J., Bingle L.E.H., Heurlier K., Pallen M.J., Penn C.W., Busby S.J.W., Hobman J.L. Gene doctoring: A method for recombineering in laboratory and pathogenic Escherichia colistrains. BMC Microbiol. 2009; 9:252. 20003185 10.1186/1471-2180-9-252 PMC2796669

[54] Baba T., Ara T., Hasegawa M., Takai Y., Okumura Y., Baba M., Datsenko K.A., Tomita M., Wanner B.L., Mori H. Construction of Escherichia coli K-12 in-frame, single-gene knockout mutants: the Keio collection. Mol. Syst. Biol. 2006; 2:2006.0008. 10.1038/msb4100050 PMC1681482 16738554

[55] Sambrook J., Russell D.W. The Inoue method for preparation and transformation of competent *E. Coli* : “Ultra-Competent” cells. CSH Protoc. 2006; 10.1101/pdb.prot3944. 22485385

[56] Wick R.R., Judd L.M., Gorrie C.L., Holt K.E. Unicycler: resolving bacterial genome assemblies from short and long sequencing reads. PLoS Comput. Biol. 2017; 13:e1005595. 28594827 10.1371/journal.pcbi.1005595 PMC5481147

[57] Yurchenko V.V., Morozov A.A., Fedorov R.A., Bakina L.G., Zgoda V.G., Tikhonova O.V. Dataset on the effects of environmentally relevant humic acid concentrations on the liver protein profile in Japanese medaka (Oryzias latipes). Data Brief. 2022; 40:107796. 35036493 10.1016/j.dib.2022.107796 PMC8749161

[58] Łobocka M.B., Rose D.J., Plunkett I.I.I.,G., Rusin M., Samojedny A., Lehnherr H., Yarmolinsky M.B., Blattner F.R. Genome of bacteriophage P1. J. Bacteriol. 2004; 186:7032. 15489417 10.1128/JB.186.21.7032-7068.2004 PMC523184

[59] Smith G.R., Kunes S.M., Schultz D.W., Taylor A., Triman K.L. Structure of chi hotspots of generalized recombination. Cell. 1981; 24:429–436. 6453653 10.1016/0092-8674(81)90333-0

[60] Dillingham M.S., Kowalczykowski S.C. RecBCD enzyme and the repair of double-stranded DNA breaks. Microbiol. Mol. Biol. Rev. 2008; 72:642–671. 19052323 10.1128/MMBR.00020-08 PMC2593567

[61] Cheng K., Wilkinson M., Chaban Y., Wigley D.B. A conformational switch in response to Chi converts RecBCD from phage destruction to DNA repair. Nat. Struct. Mol. Biol. 2020; 27:71–77. 31907455 10.1038/s41594-019-0355-2 PMC7000243

[62] Dabert P., Ehrlich S.D., Gruss A. Chi sequence protects against RecBCD degradation of DNA in vivo. Proc. Natl. Acad. Sci. U.S.A. 1992; 89:12073–12077. 1465442 10.1073/pnas.89.24.12073 PMC50700

[63] Smith G.R. How RecBCD enzyme and Chi promote DNA break repair and recombination: a molecular biologist's view. Microbiol. Mol. Biol. Rev. 2012; 76:217–228. 22688812 10.1128/MMBR.05026-11 PMC3372252

[64] Morimatsu K., Kowalczykowski S.C. RecFOR proteins load RecA protein onto gapped DNA to accelerate DNA strand exchange: a universal step of recombinational repair. Mol. Cell. 2003; 11:1337–1347. 12769856 10.1016/s1097-2765(03)00188-6

[65] Cox M.M., Goodman M.F., Keck J.L., van Oijen A., Lovett S.T., Robinson A. Generation and repair of postreplication gaps in Escherichia coli. Microbiol. Mol. Biol. Rev. 2023; 87:e00078-22. 37212693 10.1128/mmbr.00078-22 PMC10304936

[66] Köppen A., Krobitsch S., Thoms B.., Wackernagel W. Interaction with the recombination hot spot chi in vivo converts the RecBCD enzyme of Escherichia coli into a chi-independent recombinase by inactivation of the RecD subunit. Proc. Natl. Acad. Sci. U.S.A. 1995; 92:6249–6253. 7541534 10.1073/pnas.92.14.6249 PMC41495

[67] Palas K.M., Kushner S.R. Biochemical and physical characterization of exonuclease V from Escherichia coli. Comparison of the catalytic activities of the RecBC and RecBCD enzymes. J. Biol. Chem. 1990; 265:3447–3454. 2154479

[68] Salaj-Smic E., Marsić N., Trgovcević Z., Lloyd R.G. Modulation of EcoKI restriction in vivo: role of the lambda Gam protein and plasmid metabolism. J. Bacteriol. 1997; 179:1852–1856. 9068628 10.1128/jb.179.6.1852-1856.1997 PMC178906

[69] Walker G.C. Neidhardt F.C., Curtiss R.I., Ingraham J.L., Lin C.C.L., Low K.B., Magasanik B., Reznikoff W.S., Riley M., Schaechter M., Umbarger H.E. The SOS Response of Escherichia coli. Escherichia coli and Salmonella: Cellular and Molecular Biology. 1996; Washington, D.C. ASM Press 1400–1416.

[70] Maslowska K.H., Makiela-Dzbenska K., Fijalkowska I.J. The SOS system: a complex and tightly regulated response to DNA damage. Environ. Mol. Mutagen. 2019; 60:368–384. 30447030 10.1002/em.22267 PMC6590174

[71] Witkin E.M. RecA protein in the SOS response: milestones and mysteries. Biochimie. 1991; 73:133–141. 1883877 10.1016/0300-9084(91)90196-8

[72] Adikesavan A.K., Katsonis P., Marciano D.C., Lua R., Herman C., Lichtarge O. Separation of recombination and SOS response in Escherichia coli RecA suggests LexA interaction sites. PLoS Genet. 2011; 7:e1002244. 21912525 10.1371/journal.pgen.1002244 PMC3164682

[73] Thoms B., Wackernagel W. Genetic control of damage-inducible restriction alleviation in Escherichia coli K12: an SOS function not repressed by lexA. Mol. Gen. Genet. 1984; 197:297–303. 6097797 10.1007/BF00330977

[74] Smirnov G.B., Bodoev I.N., Makarova A.P., Butusova T.B., Veselovsky V.A., Gulyaev A.S., Shitikov E.A., Ilina E.N. Comparative genomics of the Escherichia coli strains АВ1157, АВ2463, АВ2494, and АВ1885. Mol. Genet. Microbiol. Virol. 2019; 34:182–187.

[75] Little J.W. Autodigestion of lexA and phage lambda repressors. Proc. Natl. Acad. Sci. U.S.A. 1984; 81:1375–1379. 6231641 10.1073/pnas.81.5.1375 PMC344836

[76] Little J.W., Edmiston S.H., Pacelli L.Z., Mount D.W. Cleavage of the Escherichia coli lexA protein by the recA protease. Proc. Natl. Acad. Sci. U.S.A. 1980; 77:3225–3229. 6447873 10.1073/pnas.77.6.3225 PMC349587

[77] Cory M.B., Li A., Hurley C.M., Hostetler Z.M., Venkatesh Y., Jones C.M., Petersson E.J., Kohli R.M. Engineered RecA constructs reveal the minimal SOS activation complex. Biochemistry. 2022; 61:2884–2896. 36473084 10.1021/acs.biochem.2c00505 PMC9982712

[78] Lin L.-L., Little J.W. Isolation and characterization of noncleavable (Ind-) mutants of the LexA repressor of Escherichia coli K-12. J. Bacteriol. 1988; 170:2163–2173. 2834329 10.1128/jb.170.5.2163-2173.1988 PMC211102

[79] Zaman M.M., Boles T.C. Plasmid recombination by the RecBCD pathway of Escherichia coli. J. Bacteriol. 1996; 178:3840–3845. 8682788 10.1128/jb.178.13.3840-3845.1996 PMC232644

[80] Sakai A., Cox M.M. RecFOR and RecOR as distinct RecA loading pathways. J. Biol. Chem. 2009; 284:3264–3272. 18986990 10.1074/jbc.M807220200 PMC2631980

[81] Bi X., Liu L.F. recA-independent and recA-dependent intramolecular plasmid recombination: differential homology requirement and distance effect. J. Mol. Biol. 1994; 235:414–423. 8289271 10.1006/jmbi.1994.1002

[82] Lovett S.T., Hurley R.L., Sutera V.A. Jr, Aubuchon R.H., Lebedeva M.A Crossing over between regions of limited homology in Escherichia coli: RecA-dependent and RecA-independent pathways. Genetics. 2002; 160:851–859. 11901106 10.1093/genetics/160.3.851 PMC1462031

[83] Buljubašić M., Hlevnjak A., Repar J., Đermić D., Filić V., Weber I., Zahradka K., Zahradka D. RecBCD-RecFOR-independent pathway of homologous recombination in Escherichia coli. DNA Repair (Amst.). 2019; 83:102670. 31378505 10.1016/j.dnarep.2019.102670

[84] Pham P., Wood E.A., Cox M.M., Goodman M.F. RecA and SSB genome-wide distribution in ssDNA gaps and ends in Escherichia coli. Nucleic Acids Res. 2023; 51:5527–5546. 37070184 10.1093/nar/gkad263 PMC10287960

[85] Jain K., Wood E.A., Romero Z.J., Cox M.M. RecA-independent recombination: Dependence on the Escherichia coli RarA protein. Mol. Microbiol. 2021; 115:1122–1137. 33247976 10.1111/mmi.14655 PMC8160026

[86] Baharoglu Z., Bikard D., Mazel D. Conjugative DNA transfer induces the bacterial SOS response and promotes antibiotic resistance development through integron activation. PLoS Genet. 2010; 6:e1001165. 20975940 10.1371/journal.pgen.1001165 PMC2958807

[87] Petrova V., Chitteni-Pattu S., Drees J.C., Inman R.B., Cox M.M. An SOS inhibitor that binds to free RecA protein: the PsiB protein. Mol. Cell. 2009; 36:121–130. 19818715 10.1016/j.molcel.2009.07.026 PMC2761248

[88] Samuel B., Burstein D. A diverse repertoire of anti-defense systems is encoded in the leading region of plasmids. 2023; bioRxiv doi:16 February 2023, preprint: not peer reviewed 10.1101/2023.02.15.528439. PMC11541004 39385022

[89] Jaffe A., Ogura T., Hiraga S. Effects of the ccd function of the F plasmid on bacterial growth. J. Bacteriol. 1985; 163:841–849. 3897195 10.1128/jb.163.3.841-849.1985 PMC219208

[90] Hall J.P.J., Wright R.C.T., Harrison E., Muddiman K.J., Wood A.J., Paterson S., Brockhurst M.A. Plasmid fitness costs are caused by specific genetic conflicts enabling resolution by compensatory mutation. PLoS Biol. 2021; 19:e3001225. 34644303 10.1371/journal.pbio.3001225 PMC8544851

[91] San Millan A., Toll-Riera M., Qi Q., MacLean R.C. Interactions between horizontally acquired genes create a fitness cost in Pseudomonas aeruginosa. Nat. Commun. 2015; 6:6845. 25897488 10.1038/ncomms7845 PMC4410645

[92] Yano H., Wegrzyn K., Loftie-Eaton W., Johnson J., Deckert G.E., Rogers L.M., Konieczny I., Top E.M. Evolved plasmid-host interactions reduce plasmid interference cost. Mol. Microbiol. 2016; 101:743–756. 27121483 10.1111/mmi.13407 PMC5024541

[93] Subramaniam S., Smith G.R. RecBCD enzyme and Chi recombination hotspots as determinants of self vs. non-self: Myths and mechanisms. Adv. Genet. 2022; 109:1–37. 36334915 10.1016/bs.adgen.2022.06.001 PMC10047805

[94] Wein T., Hülter N.F., Mizrahi I., Dagan T. Emergence of plasmid stability under non-selective conditions maintains antibiotic resistance. Nat. Commun. 2019; 10:2595. 31197163 10.1038/s41467-019-10600-7 PMC6565834

[95] Guerin É., Cambray G., Sanchez-Alberola N., Campoy S., Erill I., Da Re S., Gonzalez-Zorn B., Barbé J., Ploy M.-C., Mazel D. The SOS response controls integron recombination. Science. 2009; 324:1034. 19460999 10.1126/science.1172914

[96] Crane J.K., Alvarado C.L., Sutton M.D. Role of the SOS response in the generation of antibiotic resistance in vivo. Antimicrob. Agents Chemother. 2021; 65:e0001321. 33875437 10.1128/AAC.00013-21 PMC8373240

