# Supplementary material for: RecA-dependent or independent recombination of plasmid DNA generates a conflict with the host EcoKI immunity by launching restriction alleviation
Source: Nucleic Acids Res. 2024 Apr 3;52(9):5195–208. doi: 10.1093/nar/gkae243 (PMC11109961; doi:10.1093/nar/gkae243)
Supplement: gkae243_Supplemental_File [file gkae243_supplemental_file.pdf]

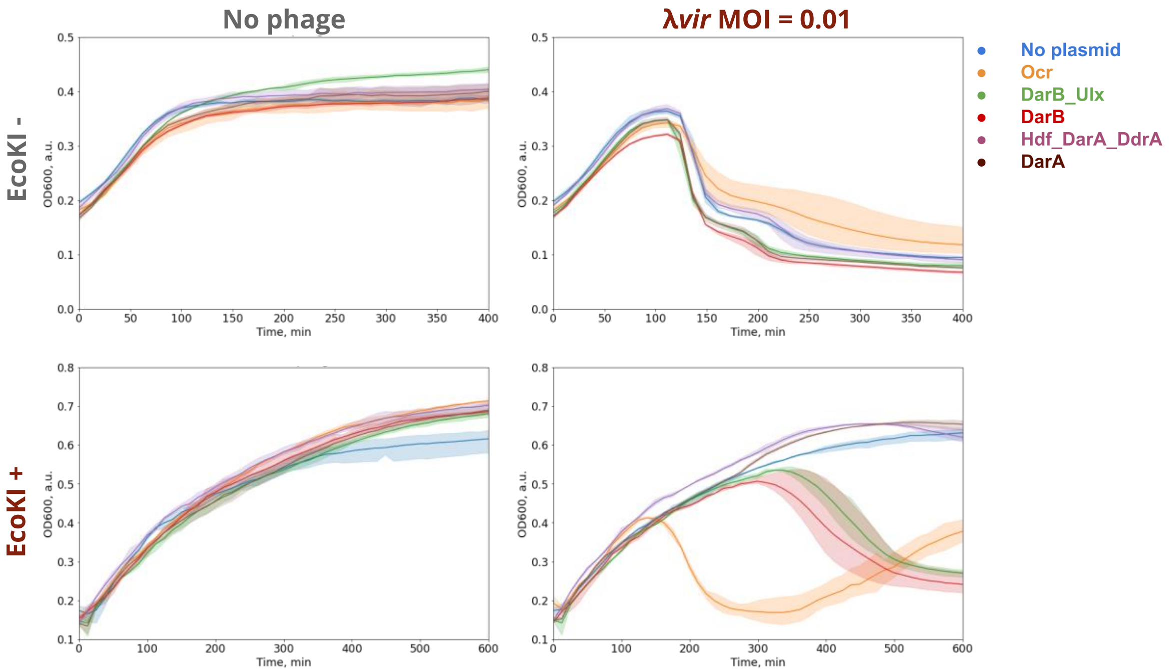

Figure S1. Growth of AB1157 (EcoKI$^+$) or AB1157*ΔhsdM* (EcoKI$^-$) liquid cultures producing EcoKI inhibitor T7 Ocr or phage P1 Dar system proteins upon infection with phage λ_{vir} at a multiplicity of infection (MOI) = 0.01. Phage was added at *t=0* to exponentially growing cultures induced with 0.2% L-arabinose 30 minutes prior to infection. Means of the three biological replicates with standard deviations are shown.

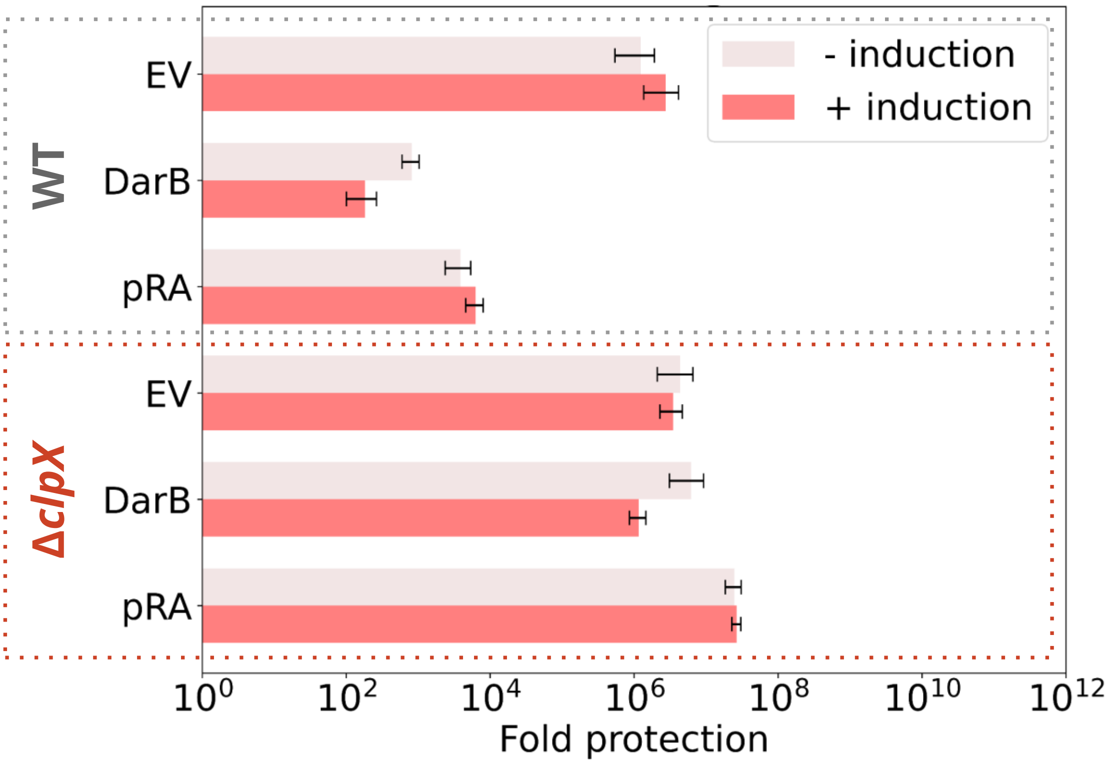

Figure S2. Induction of *darB* expression has no effect on restriction alleviation. EOP with AB1157 and AB1157 *ΔclpX* showing fold defence provided by EcoKI system against phage λ_{vir} infection in the presence of the pBAD vector, a plasmid encoding DarB, or a DarB fragment (plasmid pRA).

A

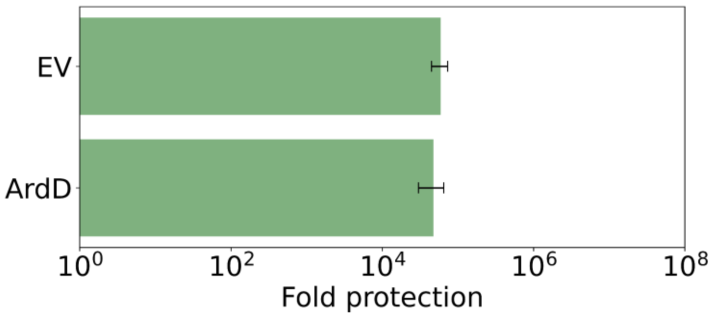

B

Transposon	GeneBank ID	Percent Identity, %	Vector	Ori	Copy number	EcoKI	Chi	Plasmid Size
pTn21	AF071413.3	100	pBR322	pMB1	Middle ~15-20	6	2	24038
pTn402	GQ857074.1	99.88	pGEM	pMB1*	High ~300-500	1	0	11267
pTn501	Z00027.1	100	pBR322	pMB1	Middle ~15-20	3	3	12721
pTn5045	FN821089.1	100	pGEM	pMB1*	High ~300-500	5	3	24897
pTn5053	L40585.1	99.97	pUC18	pMB1**	High ~500-700	2	2	10755
pTn5057	-	-	pGEM	pMB1*	High ~300-500	7	3	22101+ 10933+ 1937
pTn5060	AJ551280.1	99.98	pBR322	pMB1	Middle ~15-20	4	0	13033

pMB1* - pMB derivative origin with 1 point mutation
pMB1** - pMB derivative origin with 2 point mutations, equal to pUC18 origin

Figure S3. Anti-restriction activity of Tn5053-like non-conjugative transposons is due to plasmid-mediated RA. **A)** EOP assay with phage λ_{vir} and AB1157 carrying an empty vector (pBAD) or pBAD_*ardD*. Expression of predicted *ardD* ORF was induced with 0.2% arabinose. **B)** Features of Illumina-sequenced non-conjugative transposons and corresponding plasmid backbones. GenBank id – the closest sequence found in GenBank. pTn5057 assembly was not closed and data is provided for the sum of three largest contigs.

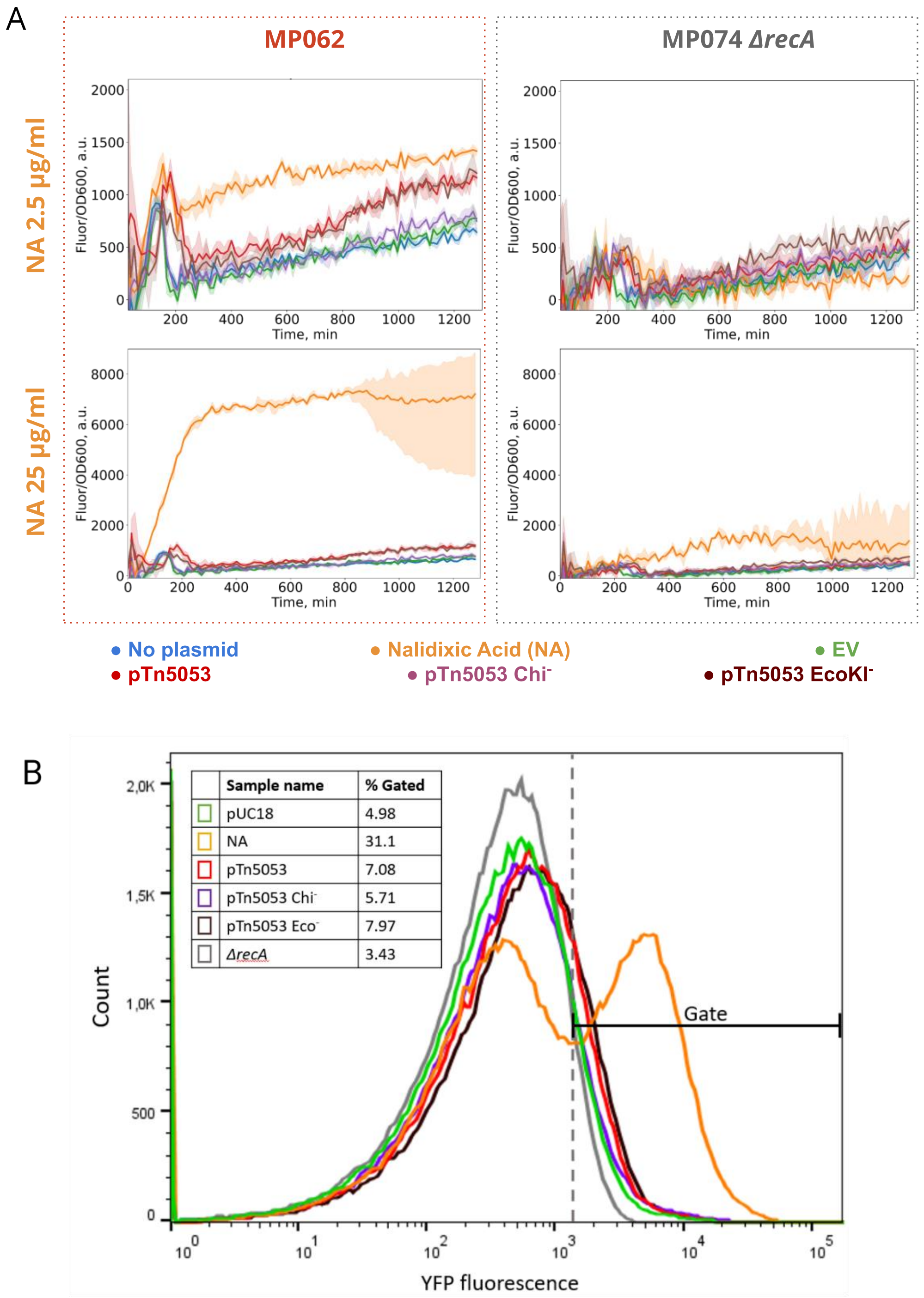

Figure S4. Chi-sites carrying plasmids induce SOS response in a subpopulation of cells. **A)** Liquid culture growth curve assay for the SOS reporter *E. coli* strain MP062 and control *ΔrecA* strain MP074 not capable of SOS response induction. Mean YFP fluorescence from three independent experiments normalized to the culture optical density (OD600) is shown with standard deviations. NA – nalidixic acid. **B)** Flow cytometry measurement of the SOS reporter *E. coli* strain MP062 carrying indicated plasmid or treated with 2.5 µg/ml nalidixic acid (NA). The proportion of population demonstrating YFP fluorescence above the indicated threshold is shown in the inset. Average values from the biological triplicate experiment are presented.

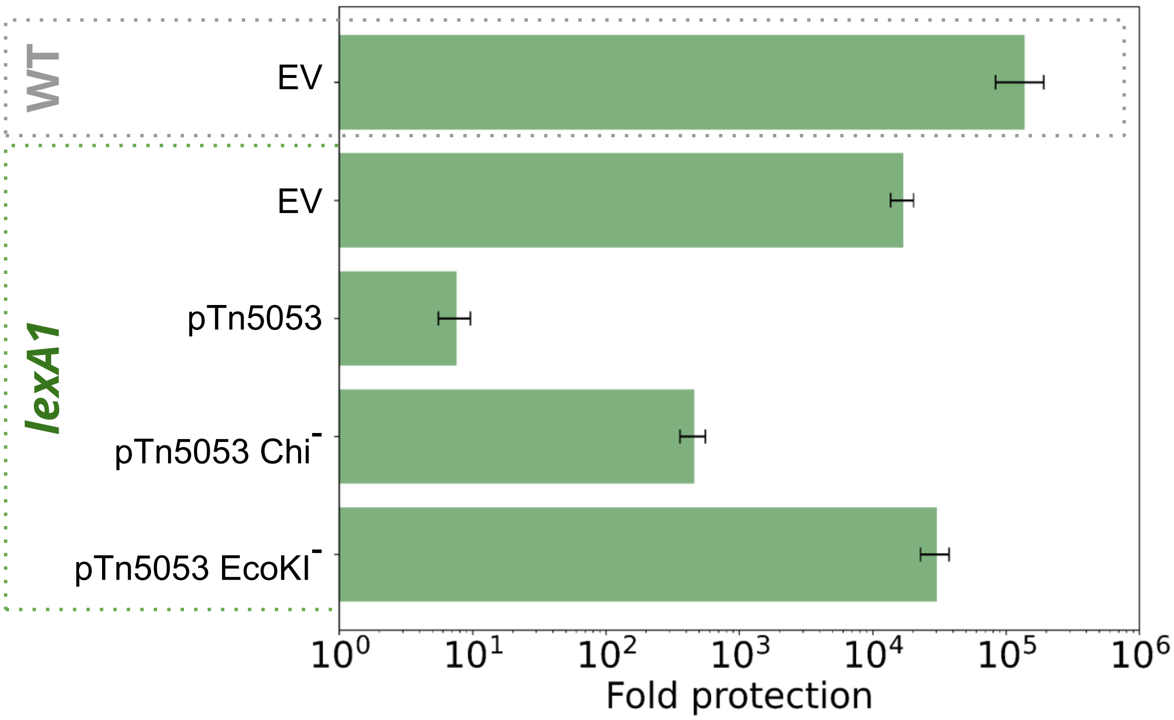

Figure S5. EOP assay with phage λ_{vir} and *lexA1* AB2494 strain carrying pUC18-Tn5053 or its Chi^{-} or EcoKI^{-} derivatives. EOP was carried in biological triplicates and diagram show mean values with standard deviation.

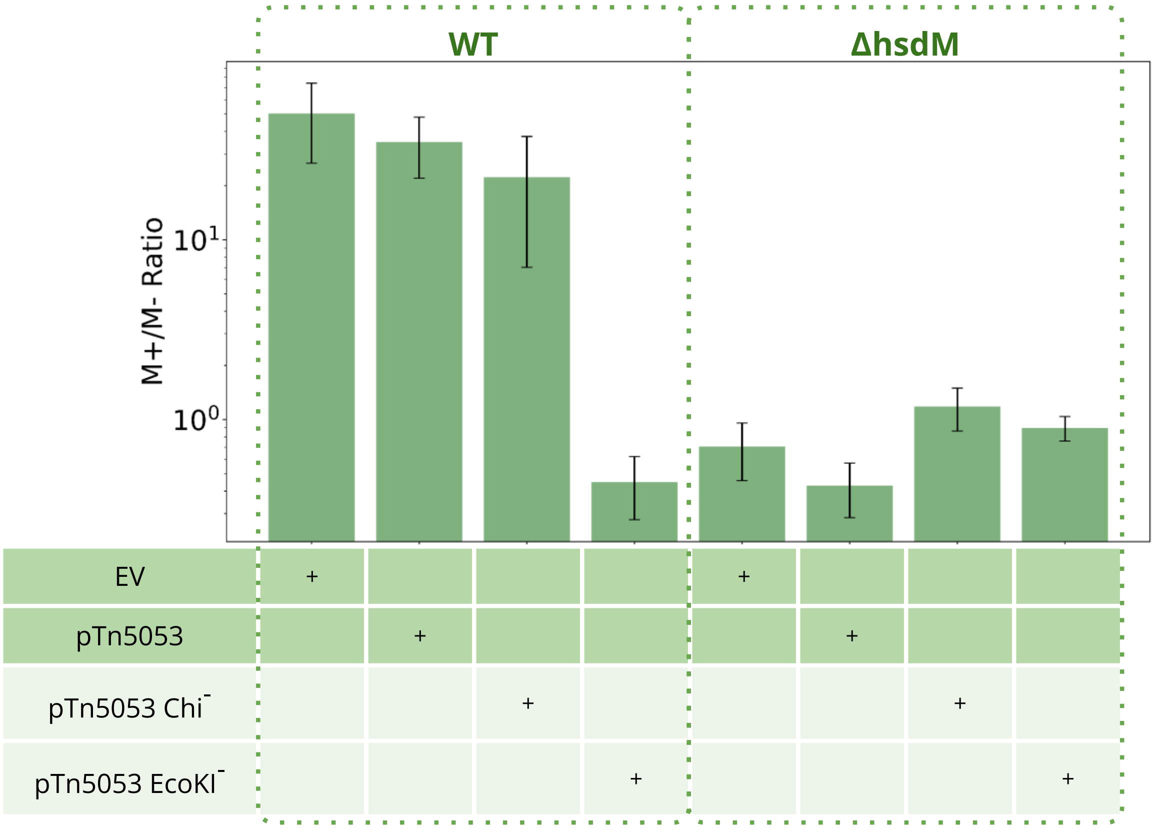

Figure S6. The presence of a Chi does not enhance plasmid *de novo* acquisition by EcoKI^{+} cells. Efficiency of transformation (EOT) assay with pUC18-Tn5053 or RA-inactive EcoKI^{-} or Chi^{-} derivatives. Chemocompetent AB1157 or AB1157*ΔhsdM* cells were transformed with non-methylated or methylated plasmids and the results are presented as a fold-change difference in efficiency of methylated plasmid transformation relative to non-methylated plasmid, which reflects efficiency of EcoKI defence.

Supplementary Table S1. Bacterial strains, plasmids, and phages used in the study.

***E. coli* strains**	**Comments**	**Source**
BW25113	*E. coli* K12 F⁻ *Δ*(*araD-araB*)567 *ΔlacZ*4787(::*rrnB*-3) λ– *rph*-1 *Δ*(*rhaD-rhaB*)568 *hsdR*514	Lab stock
AB1157	*E. coli* B F– *ompT* *hsdS*$_B$ (r_B–, m_B–) *gal dcm* (DE3)	Lab stock
XL1-Blue	*E. coli* K12 F⁻ *thr*-1 *araC*14 *leuB*6(Am) *Δ(gpt-proA)*62 *lacY*1 *tsx*-33 *qsr'*-0 *glnX*44(AS) *galK*2(Oc) λ⁻ *Rac*-0 *hisG*4(Oc) *rfbC*1 *mgl*-51 *rpoS*396(Am) *rpsL*31 *kdgK*51 *xylA*, *mtl*-1 *argE*3(Oc) *thiE*1, Str^R	Evrogen
AB1157 *ΔclpX*	AB1157 *clpX::KnR* – deletion transferred by P1 transduction from KEIO collection	This work
AB1157 *ΔhsdM*	AB1157 *hsdM::KnR* – deletion transferred by P1 transduction from KEIO collection	This work
MP062	MG1655, *ΔattHK022::(PsulA-yfp)*, *Δattλ::(PN25-tetR, PlacIq-lacI, SpR)*	(1)
MP074	MG1655, *ΔattHK022::(PsulA-yfp), Δattλ::(PN25-tetR, PlacIq-lacI, SpR), ΔrecA*	(1)
AB1157 *ΔrecA*	AB1157 *recA::KnR* – deletion transferred by P1 transduction from KEIO collection	This work
AB1157 *ΔrecB*	AB1157 *recB::KnR* – deletion transferred by P1 transduction from KEIO collection	This work
AB1157 *ΔrecC*	AB1157 *recC::KnR* – deletion transferred by P1 transduction from KEIO collection	This work
AB1157 *ΔrecD*	AB1157 *recD::KnR* – deletion transferred by P1 transduction from KEIO collection	This work
AB2494	AB1157 derivative carrying *lexA1* allele, Str^R	(2)
AB1157 *ΔrecB ΔrecF*	AB1157Δ*recB recF::KnR* constructed on the basis of AB1157Δ*recB* strain via pCP20 KnR depletion and P1 transduction of *ΔrecF*	This work
AB1157 *ΔrecB ΔrecO*	AB1157Δ*recB recO::KnR* constructed on the basis of AB1157Δ*recB* strain via pCP20 KnR depletion and P1 transduction of *ΔrecF*	This work
AB1157 *ΔhsdM Δx*	Where X - any of the above-mentioned deletions. Double deletions have been constructed on the basis of *ΔhsdM* to test their direct effect on the phage λ_{vir} infection	This work
**Phages**	**Comments**	**Source**
$P1_{vir}$		Lab stock
λ_{vir}		Lab stock
T7 *Δ0.3*	EcoKI-sensitive variant, lacks anti-restriction protein Ocr	(3)
**Plasmids**	**Comments**	**Source**
pBAD L24	A variant of pBAD-HisB, empty vector, Amp^R	(3)
pBAD Ocr wt	pBAD L24 encoding T7 Ocr, *araBAD* promoter, Amp^R	(3)
pBAD DarB_Ulx	pBAD L24 encoding P1 DarB_Ulx, *araBAD* promoter, Amp^R	This work
pBAD DarB	pBAD L24 encoding P1 DarB, *araBAD* promoter, Amp^R	This work
pBAD Ulx	pBAD L24 encoding P1 Ulx, *araBAD* promoter, Amp^R	This work
pBAD Hdf_DarA_DdrA	pBAD L24 encoding P1 Hdf_DarA_DdrA, *araBAD* promoter, Amp R	This work
pBAD Hdf	pBAD L24 encoding P1 Hdf, *araBAD* promoter, Amp^R	This work
pBAD DarA	pBAD L24 encoding P1 DarA, *araBAD* promoter, Amp^R	This work
pBAD DdrA	pBAD L24 encoding P1 DdrA, *araBAD* promoter, Amp^R	This work
pRA	pBAD L24 encoding P1 DarB first 350 N-terminal amino acids, *araBAD* promoter, Amp^R	This work
pBAD_DarB_stop	pBAD_DarB with premature stop-codon at the 9th codon of	This work

	darB, *araBAD* promoter, Amp^R	
pRA_stop	pRA with premature stop-codon at the 9th codon of *darB*,, *araBAD* promoter, Amp^R	This work
pRA Chi$^-$	pRA with Chi site synonymous mutation CTGGTG → TTAGTT in *darB*, *araBAD* promoter, Amp^R	This work
pRA_EcoKI$^-$	pRA with EcoKI site synonymous mutation GCACGAGTGGGTT → GCCCGAGTGGGCT in *bla* gene, *araBAD* promoter, Amp^R	This work
pRA_Chi_rev	pRA_Chi$^-$ with Chi site in reverse orientation, *araBAD* promoter, Amp^R	This work
pBAD_*ardD*	pBAD encoding ORF of a predicted *ardD* gene, *araBAD* promoter, Amp^R	This work
pUC-18	Empty vector	Lab Stock
pTn5053	pUC-18 derivative with Tn5053 sequence, Amp^R	Dr. Olga Melkina
pTn5053_*ardD*_stop	pTn5053 with premature stop-codon at the 11th codon of hypothetical gene *ardD*, Amp^R	This work
pTn5053_*ΔardD*	pTn5053 based plasmid with deletion of hypothetical gene *ardD*, Amp^R	This work
pTn5053 Chi^{-1}	pTn5053 with synonymous mutations GCTGGTGG→GTTAGTTG in *tniB* gene, Amp^R	This work
pTn5053 Chi^{-2}	pTn5053 with synonymous mutations of Chi site GCTGGTGG→GTTAGTTG in *merT* gene, Amp^R	This work
pTn5053 Chi$^-$	pTn5053 with synonymous mutations of Chi sites in *tniB* and *merT*, Amp^R	This work
pTn5053 EcoKI^{-1}	pTn5053 with EcoKI site synonymous mutation GCACNNNNNNNGTT→GCGCNNNNNNNGCT in *merR* gene, Amp^R	This work
pTn5053 EcoKI^{-2}	pTn5053 with EcoKI site synonymous mutation GCACNNNNNNNGTT→GCCCNNNNNNNGGT in *bla* gene, Amp^R	This work
pTn5053 EcoKI$^-$	pTn5053 with synonymous mutations of EcoKI sites in *merR* and *ampR*, Amp^R	This work
pTn5053 Chi^{-1} EcoKI^{-1}	pTn5053 with synonymous mutations of Chi site in *tniB* and EcoKI site in *merR*, Amp^R	This work
pTn5053 Chi^{-1} EcoKI^{-2}	pTn5053 with synonymous mutations of Chi site in *tniB* and EcoKI site in *bla*, Amp^R	This work
pTn5053 Chi^{-2} EcoKI^{-1}	pTn5053 with synonymous mutations of Chi site in *merT* and EcoKI site in *merR*, Amp^R	This work
pTn5053 Chi^{-2} EcoKI^{-2}	pTn5053 with synonymous mutations of Chi site in *merT* and EcoKI site in *bla*, Amp^R	This work
pACBSR	Plasmid from gene doctoring system, encodes I-SceI and *λ* Red + Gam under control of *araBAD* promoter, Cm^R	(4)
pACBSR Red-Gam	pACBSR with deletion of I-SceI gene, *λ* Red + Gam are controlled by *araBAD* promoter, Cm^R	This work
pACBSR Gam	pACBSR Red-Gam with deletion of *λ* Red, *λ* Gam is controlled by *araBAD* promoter, Cm^R	This work
pACBSR Red	pACBSR Red-Gam with deletion of *λ* Gam, *λ* Red is controlled by *araBAD* promoter, Cm^R	This work
pTn21	pBR322 derivative with Tn21 sequence, Amp^R	Dr. Olga Melkina
pTn402	pGEM derivative with Tn402 sequence, Amp^R	Dr. Olga Melkina
pTn501	pBR322 derivative with Tn501 sequence, Amp^R	Dr. Olga Melkina
pTn5045	pGEM derivative with Tn5045 sequence, Amp^R	Dr. Olga Melkina
pTn5057	pGEM derivative with Tn5057 sequence, Amp^R	Dr. Olga Melkina

pTn5060	pBR322 derivative with Tn5060 sequence, Amp^R	Dr. Olga Melkina
pTG	Empty vector, Cm^R	(5)
pCP20	FLP1, *λ* cI857$^+$, *λ* p_R Rep^{ts}; Amp^R, Cm^R	(6)

Supplementary Table S2. Primers used in the study.

**Name**	**Sequence (5′->3′)**	**Purpose**
pBAD_For	ATGCCATAGCATTTTTATCC	**Sanger sequencing of pBAD**
pBAD_Rev	GATTTAATCTGTATCAGGCTG	
L24_darB_ulx_F	TTTTTGGGCTAACAGGAGGAAGAAT ATGAACAAGCTATCTATGGG	**Cloning of P1 *dar* genes**
pBAD_darB_ulx_R	AAGCTTGCGGCCGCGAGCTCCATG ATCAAGTTTTCTCTCCAGC	
L24_darB_R	CAGCCAAGCTTGCGGCCGCGAGCT CTTATGCGTATTGTTGGATGA	
DarB_check_R	ATGTAACAGCTGGTTTATAC	
L24_ulx_F	TTTGGGCTAACAGGAGGAAGAATT CATGGCCACACTATCTGATAC	
Ulx_check_F	GATTACTGAAAAATCCCCG	
pBAD_hdf_darA_ddrA_F	TTTTTGGGCTAACAGGAGGAAGAAT ATGTGTCAGATGACGAAAAA	
pBAD_ hdf_darA_ddrA_R	AAGCTTGCGGCCGCGAGCTCCATG ATTATGCTGCCTCTTTAATGT	
hdf_check_R	GATCTGCTGTTGATTGAAAG	
ddrA_check_F	GACCTCAGCGATAAGTTG	
L24_ hdf_R	CAGCCAAGCTTGCGGCCGCGAGCT CTTAATTTCCTGCTTTCAGTTG	
L24_darA_F	TTTGGGCTAACAGGAGGAAGAATT CATGGAACAGTTCAATATCAAT	
L24_ darA_R	CAGCCAAGCTTGCGGCCGCGAGCT CTCATGCTGCTACCCCC	
DarA_check_R	GGCTATGAAACAGAATGC	
L24_DdrA_F	TTTGGGCTAACAGGAGGAAGAATT CATGACACTATCAGCTATTG	
L24_darB_1-350_R	CAGCCAAGCTTGCGGCCGCGAGCT CTTAGCTTTCGAAACAATG	
DarB_stop_F	TAATTTCGCTGTTCAAGTGTC	**Introduction of pre-mature stop codon into *darB***
DarB_stop_R	CACCCCCATAGATAGCTTG	
pRA_Chi_F	TTAGTTGACGGCGCGACACTGAC	**Mutations of Chi and EcoKI sites in pRA**
pRA_Chi_R	CGAATTAAGCAAATCTATCGCCTTC	
pRA_EcoKI_F	TGGGCTACATCGAACTGGATCTCAA C	
pRA_EcoKI_R	CTCGGGCACCCAACTGATCTTCAG	

pRA_Chi_rev_F	CAGCACGGCGCGACACTGACAG	
pRA_Chi_rev_R	GTGGGAATTAAGCAAATCTATCGCC TTCTGATTC	
pBAD_ArdD_For	CATACCCGTTTTTTTGGGCAGGAGA ACCCAAATGGGACAAGG	**Cloning, mutation or deletion of *ardD***
pBAD_ArdD_Rev	TACCGAGCTCGAATTCGCTAGTTAC CGGGGACGAC	
UC_ArdD_b1	TGTCCCATCCGGCCGTC	
UC_ArdD_b2	TTCGTCGTGGATCATCTGCATC	
ArdD_stop_F	TCAGCACTATTTAGAGATCCACTAC CG	
ArdD_stop_R	CCTTCCGGTTCCAGTACC	
V3_ArdD_Chi_1_R	TCGCCGATGGTGCCTTC	**Mutations of Chi and EcoKI sites in pTn5053**
V3_ArdD_Chi_1_F	GCTTGCACATCTTCTTGTGG	
ArdD_Chi_1check_F	CGAGCCGATGCTGCTGCC	
ArdD_Chi_1check_R	GAGCCATGAGGACAGGGC	
V3_ArdD_Chi_2_F	AGCCACGCCGATAAAGATGG	
V3_ArdD_Chi_2_R	CTTGTGGCGTTGTTCTTCG	
ArdD_Chi_2check_F	GGCACGACGTAGGGAAATCC	
ArdD_Chi_2check_R	CGCGGCGCGCTCTTCAC	
Tn_EcoKI_1_F	CAGCGCGGACGCGGTGTAG	
Tn_EcoKI_1_R	CGCGGCTCTTTCTTCCCTTG	
UC_ArdD_b2	GATGCAGATGATCCACGACGAA	
dClpx_check_F	ACTGTGCCGCTATACTTATC	**Verification of chromosomal gene deletions**
dClpx_check_R	GCTTCACGCCATCTATTAAC	
RecA_check_F	TGATTCTGTCATGGCATATC	
RecA_check_R	CACAAGGTCTGCAATGCATA	
RecB_dir	CGCGTTGCAGCAAACAATGC	
RecB_rev	CCACAGCTTCCAGTAATTGC	
RecC_dir	CTGCATTGCCCGAATCGTC	
RecC_rev	AAAGTAAGCGGATAGATTGCGC	
RecD_dir	CCCTGATGGATGAGATGTTTG	
RecD_rev	AACACTCGTACGTCGCATCC	

recF_check_F	TTTGATACTGGAGGAGTCAT	
recF_check_R	ATGCGGCCAGCCAGAGCGCG	
recO_check_F	TGCGCAGCGTAGCGATATGG	
recO_check_R	AGTCTCGGTTACGTTGACGA	
F_dISce	GGGCCCGACGTCTGCAG	**Construction of pACBSR derivatives**
R_dISce	ATAGGTACCCTCCTGCTAGCCC	
R_ACBSR_2.1	CCTGAAATAGCTGTGAAAATATCG	
F_dGam	AACGAATGAGTACTGCACTC	
R_dGam	TTTTTATAACCTCCTTAGCATG	
R_ACBSR_2.2	ACCTTTAAATGCCGTCTGG	
F_dExo_bet	CGCATCCTCACGATAATATCC	
R_dExo_bet	TCGTTTTATACCTCTGAATCAATATC	

Supplementary references

1. Pleška,M., Qian,L., Okura,R., Bergmiller,T., Wakamoto,Y., Kussell,E. and Guet,C.C. (2016) Bacterial autoimmunity due to a restriction-modification system. *Current Biology*, **26**, 404–409.
2. Smirnov,G.B., Bodoev,I.N., Makarova,A.P., Butusova,T.B., Veselovsky,V.A., Gulyaev,A.S., Shitikov,E.A. and Ilina,E.N. (2019) Comparative Genomics of the Escherichia coli Strains AB1157, AB2463, AB2494, and AB1885. *Molecular Genetics, Microbiology and Virology*, **34**, 182–187.
3. Isaev,A., Drobiazko,A., Sierro,N., Gordeeva,J., Yosef,I., Qimron,U., Ivanov,N. V and Severinov,K. (2020) Phage T7 DNA mimic protein Ocr is a potent inhibitor of BREX defence. *Nucleic Acids Res*, **48**, 5397–5406.
4. Lee,D.J., Bingle,L.E.H., Heurlier,K., Pallen,M.J., Penn,C.W., Busby,S.J.W. and Hobman,J.L. (2009) Gene doctoring: a method for recombineering in laboratory and pathogenic Escherichia colistrains. *BMC Microbiol*, **9**, 1–14.
5. Gordeeva,J., Morozova,N., Sierro,N., Isaev,A., Sinkunas,T., Tsvetkova,K., Matlashov,M., Truncaitė,L., Morgan,R.D. and Ivanov,N. V (2019) BREX system of Escherichia coli distinguishes self from non-self by methylation of a specific DNA site. *Nucleic Acids Res*, **47**, 253–265.
6. Cherepanov,P.P. and Wackernagel,W. (1995) Gene disruption in Escherichia coli: TcR and KmR cassettes with the option of Flp-catalyzed excision of the antibiotic-resistance determinant. *Gene*, **158**, 9–14.
